# Expression analysis of asthma candidate genes during human and murine lung development

**DOI:** 10.1186/1465-9921-12-86

**Published:** 2011-06-23

**Authors:** Erik Melén, Alvin T Kho, Sunita Sharma, Roger Gaedigk, J Steven Leeder, Thomas J Mariani, Vincent J Carey, Scott T Weiss, Kelan G Tantisira

**Affiliations:** 1 Channing Laboratory, Brigham and Women's Hospital and Harvard Medical School, Boston, MA, USA; 2 Institute of Environmental Medicine, Karolinska Institutet, Stockholm, Sweden; 3 Astrid Lindgren Children's Hospital, Karolinska University Hospital, Stockholm, Sweden; 4 Children's Hospital Informatics Program, Boston, MA, USA; 5 Division of Pulmonary and Critical Care Medicine, Brigham and Women's Hospital, Boston, MA, USA; 6 Division of Clinical Pharmacology and Medical Toxicology, Department of Pediatrics, Children's Mercy Hospitals and Clinics, Kansas City, MO, USA; 7 Division of Neonatology and Center for Pediatric Biomedical Research, University of Rochester, Rochester NY, USA; 8 Partners Center for Personalized Genetic Medicine, Boston, MA, USA

**Keywords:** Asthma, Development, Expression, Genetics, Lung

## Abstract

**Background:**

Little is known about the role of most asthma susceptibility genes during human lung development. Genetic determinants for normal lung development are not only important early in life, but also for later lung function.

**Objective:**

To investigate the role of expression patterns of well-defined asthma susceptibility genes during human and murine lung development. We hypothesized that genes influencing normal airways development would be over-represented by genes associated with asthma.

**Methods:**

Asthma genes were first identified via comprehensive search of the current literature. Next, we analyzed their expression patterns in the developing human lung during the pseudoglandular (gestational age, 7-16 weeks) and canalicular (17-26 weeks) stages of development, and in the complete developing lung time series of 3 mouse strains: A/J, SW, C57BL6.

**Results:**

In total, 96 genes with association to asthma in at least two human populations were identified in the literature. Overall, there was no significant over-representation of the asthma genes among genes differentially expressed during lung development, although trends were seen in the human (Odds ratio, OR 1.22, confidence interval, CI 0.90-1.62) and C57BL6 mouse (OR 1.41, CI 0.92-2.11) data. However, differential expression of some asthma genes was consistent in both developing human and murine lung, e.g. *NOD1, EDN1, CCL5, RORA *and *HLA-G*. Among the asthma genes identified in genome wide association studies, *ROBO1*, *RORA, HLA-DQB1, IL2RB *and *PDE10A *were differentially expressed during human lung development.

**Conclusions:**

Our data provide insight about the role of asthma susceptibility genes during lung development and suggest common mechanisms underlying lung morphogenesis and pathogenesis of respiratory diseases.

## Introduction

There is good evidence that genetic factors strongly influence the risk of asthma, and associations between numerous genes and asthma have been evaluated in the past decades [1,2]. Recent genome wide association studies (GWAS) of asthma have identified several additional asthma susceptibility genes [3-10]. Little is known about the role of most asthma susceptibility genes during human lung development.

The "developmental origins" hypothesis [11] proposes that specific *in utero *events at critical periods during organogenesis and maturation result in long-term physiological or metabolic changes, ultimately contributing to disease in later life [12,13]. Our group previously showed that *Wnt *signaling genes that were differentially expressed during fetal lung development were associated with impaired lung function in two cohorts of school-aged asthmatic children [14]. These results suggest the importance of early life events in determining lung function. They also highlight the benefit of integrating gene expression and genetic association data to connect transcriptomic events in the early developing lung to genetic associations of lung function in later life.

Asthma is a disease characterized by both airway inflammation and smooth muscle contraction, leading to airway obstruction. Dendritic cells, mast cells, and T-lymphocytes, as well as airway smooth muscle cells, all begin to appear within the lung parenchyma during the pseudoglandular stage of lung development. We therefore hypothesized that genes influencing normal airways development, especially during the branching morphogenesis stage of human lung development, would be over-represented by genes associated with asthma. To test this hypothesis, we investigated the role of a well-defined set of asthma susceptibility genes during human and murine lung development. 96 asthma genes were first identified via comprehensive search of the current literature. Next, we analyzed their expression patterns in the developing human lung during the pseudoglandular (gestational age, 7-16 weeks) and canalicular (17-27 weeks) stages of development, and in the complete developing lung time series of 3 mouse strains: A/J, SW and C57BL6.

We show that overall, there was no over-representation of the asthma genes among genes differentially expressed during lung development, which may reflect the diverse ontological contexts of the asthma genes. However, some genes showed a consistent pattern of differential expression in all developing lung data sets, e.g. *NOD1, EDN1, RORA, CCL5 *and *HLA-G*, which suggests that these genes play a fundamental role in normal lung development.

## Methods

### Tissue samples

The human fetal lung tissues were obtained from National Institute of Child Health and Human Development supported tissue databases and microarray profiled as previously described [14,15]. Creation of the tissue repository was approved by the University of Missouri-Kansas City Pediatric Institutional Review Board. 38 RNA samples from 38 subjects (estimated gestational age 7-22 weeks or 53-154 days post conception) were included in the analysis (Table 1). The murine data have previously been described and their microarray data are available at NCBI Gene Expression Omnibus (GEO, http://www.ncbi.nlm.nih.gov/geo); A/J [16], n = 24 samples; SW [17], n = 11; and C57BL6 mice [18], n = 5, Table 1.

**Table 1:** Summary characteristics of included human and murine lung data sets

Data sets	Developmental period	N samples	Platform	Probes represented on chip	Genes represented on chip	Number of asthma genes*	Number of asthma probes	Ref
Human lungs	7-22 weeks prenatal	38	Affy U133 Plus 2.0	54,675	19,501	96	220	[15]
Mouse A/J lungs	14 days prenatal - 4 weeks postnatal	24	Affy Mu74Av2	12,488	9,060	66	89	[16]
Mouse SW lungs	12 days prenatal - 4 weeks postnatal	11	Affy Mu11K A and B	13,179	7,660	60	86	[17]
Mouse C57BL6 lungs	11.5 days prenatal - 5 days postnatal	5	Affy Mouse 430 2.0	45,101	21,141	88	142	[18]

* *CCL26, GSDMB, HLA-DQB1, PTPRD, TLR10 *and *WDR36 *do not have a mouse orthologue gene.

### Microarray analysis

The developing human lung time series data is available at NCBI Gene Expression Omnibus (GEO, http://www.ncbi.nlm.nih.gov/geo), GSE14334 (Affymetrix Human Genome GeneChip U133 Plus 2.0 microarray platform). Expression values were extracted and normalized from .CEL files using the *Affy *package and the Robust Multi-array Average (RMA) method in R/BioConductor (http://www.bioconductor.org) which returns the measured expression signal of each micrroarray gene probe in logarithmic base 2 scale. Validation of the human microarray analysis by qPCR for genes differentially expressed during lung development has been performed earlier and this demonstrated that 83% of individual gene expression trajectories could be replicated [15]. The developing whole mouse lung transcriptome data from three different mouse strains were extracted and normalized, separately, using RMA in R/BioConductor; 24 samples from A/J (Affymetrix Mu74Av2 platform); 11 samples from SW (Affymetrix Mu11K A and B platforms); and 5 samples from C57BL6 (Affymetrix Mouse 430 Plus 2.0 platform).

### Literature search

A PubMed (http://www.ncbi.nlm.nih.gov/pubmed) search was performed on March 8, 2010 using the terms 1) "asthma" together with 2) "genetic association" or "case control" in order to cover all published papers between July 1, 2008 and December 31, 2009. We applied the following inclusion criteria for an asthma gene: 1) significant association with asthma affection status in at least two populations and 2) at least one significant association study with no fewer than 150 cases and 150 controls or 150 trios. Genes identified through three earlier literature searches based on papers published before July 1, 2008 were also included if they met our two predefined criteria [1,19,20]. In addition, all GWAS of asthma published through September 2010, were also evaluated and asthma genes were included if our criteria were met. Please see Supplemental data for details about the asthma genes included in our analyses. Mouse orthologues of human genes were identified using NCBI's HomoloGene database (http://www.ncbi.nlm.nih.gov/homologene).

### Statistical analysis

Differential gene expression analysis relative to gestational age was performed using a linear regression model (lmFit) as implemented in the *Limma *package in R/BioConductor. Each microarray gene probe's logarithmic base 2 expression signal was regressed against the gestational age as a continuous variable representing days of the developing lung. We adjusted for multiple testing using the Benjamini and Hochberg method, which controls the false discovery rate (i.e. the expected proportion of false discoveries amongst the rejected hypotheses), and the adjusted p-values were used to declare a significant gene expression pattern over age [21]. "Differentially expressed" refers to an adjusted p-value of <0.05 in the linear regression model. Fisher's exact test was next performed in Stata Statistical Software (Collage Station, Tx) to test whether microrarray probes representing predefined asthma genes were over-represented among differentially expressed probes relative to probes representing "non-asthma genes". This analysis was restricted to microarray probes that were gene annotated because the asthma gene probes were all annotated. The same analysis steps were performed in human and murine data sets. Gene ontology (GO) enrichment analysis was performed using DAVID (The Database for Annotation, Visualization and Integrated Discovery) [22,23].

## Results

In total, 96 asthma susceptibility genes were identified in the literature (Additional file 1, Table E1 [1,3-10,24-96]). All genes show significant association with asthma in at least two human populations, one of which has no fewer than 150 cases and 150 controls or 150 trios. The 96 genes were represented by 220 probes on the human microarray (Table 1). Not all human genes have a mouse orthologue and the mouse microarray data sets have slightly lower numbers of asthma genes and their corresponding microarray probes.

We found that 28% of all microarray probes in the human data set were differentially expressed during the analyzed lung development period (human estimated gestational age 7-22 weeks), Table 2. A similar figure was seen in the A/J mouse and somewhat lower figures in the SW and C57BL6 mouse strains. Gene ontology (GO) enrichment analysis using DAVID of the human list of differentially expressed genes returned 879 significant GO terms, of which 6 terms pertain directly to the lung development. Among the asthma gene probes, 32% were differentially expressed during early human lung development. While there was a trend towards over-representation (Odds ratio, OR 1.22, CI 0.90-1.62) this was not statistically significant in comparison to the non-asthma gene probes (28%). In agreement with the human data, no over-representation of asthma gene probes was found among probes differentially expressed during lung development in mice strains, although there was a trend in the C57BL6 strain (OR 1.41, CI 0.92-2.11), Table 2.

**Table 2:** Proportion of the asthma gene probes among probes differentially expressed during lung development in human and mouse data sets

	Human lungs			Mouse A/J lungs			Mouse SW lungs			Mouse C57BL6 lungs		
	Asthma probes			Asthma probes			Asthma probes			Asthma probes		
Differentially expressed	Yes	No	Total*	Yes	No	Total*	Yes	No	Total*	Yes	No	Total*
Yes	71	11373	11444	25	3285	3310	15	2052	2067	32	6637	6669
No	149	29017	29166	64	8511	8575	71	9189	9260	110	32161	32271
Total	220	40390	40610	89	11796	11885	86	11241	11327	142	38798	38940
												
% differentially expressed probes	32	28	28	28	28	28	17	18	18	23	17	17
p-value†	0.18			1.0			1.0			0.09		
OR (95% CI)	1.22 (0.90-1.63)			1.01 (0.61-1.63)			0.95 (0.50-1.67)			1.41 (0.92-2.11)		

* Analyses restricted to probes represented by annotated genes.

† Fisher's exact test.

"Differentially expressed" refers to an adjusted p-value of <0.05 in the linear regression model.

OR = Odds ratio. CI = Confidence interval.

Although asthma genes as a group was not differentially expressed more than non-asthma genes during early lung development, some genes were consistently differential expressed, as listed in Table 3 (see full list in Additional file  1, Table E2). Expression of *NOD1, EDN1 *and *IL4R *were positively correlated with gestational age in the human data, whereas *ROBO1 *and *PLAUR *were negatively correlated (i.e. lower expression levels the higher gestational age). Among the asthma genes identified in GWAS, *ROBO1, RORA, HLA-DQB1, IL2RB *and *PDE10A *showed most significant evidence of involvement in lung development (all adjusted p < 0.001 for differential expression). Analyses were also done comparing gene expression patterns between the pseudoglandular (primary branching morphogenesis stage) and canalicular stages (with 112 days post conception as the dividing time point between the 2 stages). The list of top genes differentially expressed between these two stages (Additional file  1, Table E3 and Figure E1) corresponds well with the list of top genes using time as a continuous variable (Table 3).

**Table 3:** Gene expression analysis of specific asthma genes and evidence for differential expression during human lung development (adjusted p < 0.001 cut off)

Human gene symbol	Probe id	Average expression	Adjusted p-value for differential expression	Beta coefficient†
*NOD1*	221073_s_at	7.6	7.0E-8	0.012
*EDN1*	222802_at	9.3	1.6E-6	0.026
*EDN1*	218995_s_at	8.6	4.4E-6	0.019
*ROBO1*	213194_at	10.2	1.5E-5	-0.009
*IL4R*	203233_at	7.3	3.3E-5	0.010
*RORA*	226682_at	8.2	3.5E-5	0.022
*RORA*	236266_at	5.2	5.2E-5	0.011
*HPCAL1*	212552_at	9.4	5.4E-5	0.012
*HLA-DQB1*	212998_x_at	4.8	1.5E-4	0.012
*PLAUR*	210845_s_at	5.9	2.0E-4	-0.008
*IL2RB*	205291_at	6.5	2.5E-4	0.007
*CCL5*	204655_at	4.7	2.6E-4	0.008
*HPCAL1*	205462_s_at	7.3	4.6E-4	0.013
*TLR10*	223751_x_at	4.1	4.9E-4	0.005
*PDE10A*	205501_at	6.4	6.3E-4	-0.011
*CCL5*	1405_i_at	4.1	9.9E-4	0.008

* Adjusted p-value (B-H method) for differential expression over time.

† The beta coefficient corresponds to the mean change in gene expression per day during the studied period (7-22 weeks of gestational age).

**Figure 1:**
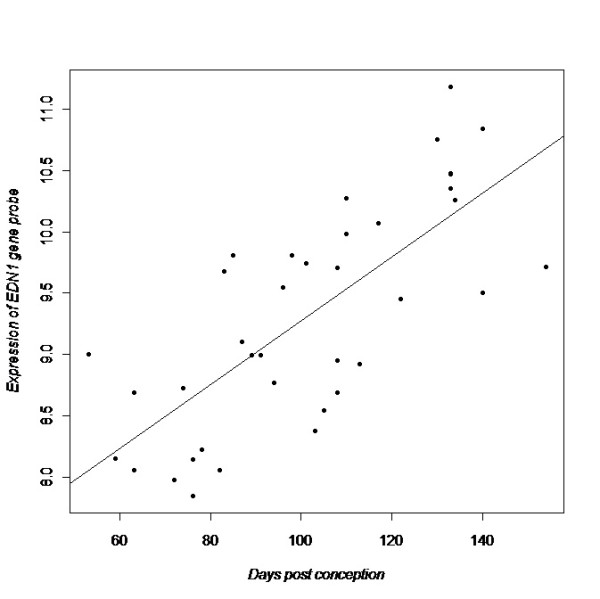
**Expression of *EDN1 *over time in human lung tissue in relation to time (days post conception), p = 1.6E-6 for differential expression**. The fitted line through the data represents the beta coefficient from linear regression analysis.

**Figure 2:**
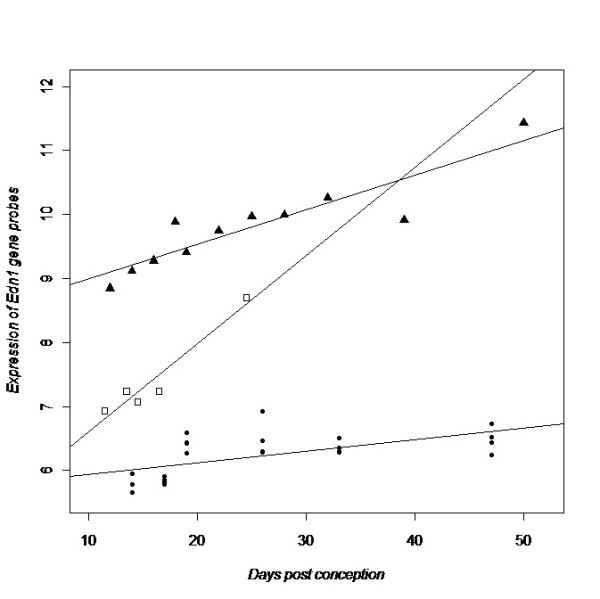
**Expression of *Edn1 *over time in mouse whole lung tissue in relation to time (days post conception)**. Solid circles represent the A/J data (p = 0.007 for differential expression), open squares represent the C57BL6 data (p = 0.02) and solid triangles represent the SW data (p = 0.001). The fitted line through each data set represents the beta coefficient from linear regression analysis.

Next, we evaluated all differentially expressed asthma genes in the human data set to see which genes showed a consistent expression pattern across human and murine data sets. Table 4 shows all genes with at least one significant probe per gene in the human data and at least one significant probe in a mouse data set (n = 19 with adjusted p-value <0.05). Eight genes had one or more significant probes in all data sets, with *NOD1, EDN1, CCL5, RORA *and *HLA-G *showing the most consistent expression patterns across human and mouse (see detailed *EDN1/Edn1 *expression over time in human and mouse lung tissue; Figure 1 and 2). In terms of bio-ontologic enrichment, the 19 asthma genes consistently differentially expressed in human and mouse lung development were enriched for ontological attributes "Regulation of cytokine production" (*IRAK3, CD86, NOD1, TNF, IL18, SCGB1A1*) and "Regulation of cell activation" (*STAT6, CD86, IL18, IL4R, RORA, SCGB1A1*) (Additional file  1, Table E4.) In terms of gene product characteristics, "Disulfide bond", "Secreted" and "Signal peptide" are attributes of a majority of the genes. 15 of the 19 genes in Table 4 have been extensively studied in human and murine experiments that support their involvement in asthma pathogenesis (Additional file  1, Table E5).

**Table 4:** Genes with at least one significant probe per gene in the human data and at least in one mouse data set (adjusted p-value <0.05)

Human gene symbol	Mouse gene symbol	Beta coef.* human	Beta coef.* A/J	Beta coef.* SW	Beta coef.* C57BL6	p-value human data*	p-value A/J†	p-value SW†	p-value C57BL6†	Combined p-value‡
***NOD1***	***Nod1***	**0.012**	-	-	**0.133**	**7.0E-8**	-	-	**2.1E-2**	**4.7E-9**
***EDN1***	***Edn1***	**0.026**	**0.018**	**0.054**	**0.138**	**1.6E-6**	**7.4E-3**	**1.4E-3**	**2.4E-2**	**3.9E-11**
*ROBO1*	*Robo1*	-0.009	-0.014	-	-0.055	1.5E-5	3.8E-2	-	9.4E-2	6.9E-7
*IL4R*	*Il4ra*	0.01	0.033	0.032	0.1	3.4E-5	3.0E-5	2.3E-2	5.5E-2	5.8E-11
***RORA***	***Rora***	**0.022**	**0.01**	**0.025**	**0.107**	**3.5E-5**	**1.3E-2**	**9.2E-3**	**2.1E-2**	**5.5E-9**
*PLAUR*	*Plaur*	-0.008	0.013	0.015	0.106	2.0E-4	2.3E-2	5.2E-2	2.1E-2	9.8E-1
***CCL5***	***Ccl5***	**0.008**	**0.022**	**0.027**	**0.079**	**2.6E-4**	**1.6E-7**	**1.9E-3**	**2.0E-2**	**5.5E-13**
***IRAK3***	***Irak3***	**-0.011**	-	-	**0.073**	**1.3E-3**	-	-	**4.5E-2**	**2.0E-2**
*IL18*	*Il18*	0.004	0.01	0.018	0.139	1.6E-3	4.4E-4	2.4E-2	2.7E-1	1.3E-7
*STAT6*	*Stat6*	0.007	0.021	0.002	0.106	1.8E-3	1.6E-2	6.0E-1	4.7E-2	2.5E-5
*CHIA*	*Chia*	0.009	0.077	0.039	0.288	1.8E-3	5.7E-3	3.4E-1	3.3E-2	4.0E-6
***HLA-G***	***H2-M3***	**0.01**	**0.021**	**0.032**	**0.103**	**2.6E-3**	**2.9E-5**	**9.2E-4**	**1.7E-2**	**3.7E-10**
*CD86*	*Cd86*	0.003	0.001	0.008	0.097	5.6E-3	9.3E-1	1.3E-1	3.1E-2	1.9E-3
*PRNP*	*Prnp*	-0.003	0.02	0.031	0.106	8.9E-3	8.8E-3	1.1E-1	7.2E-2	5.0E-1
***PCDH1***	***Pcdh1***	**0.006**	-	-	**0.232**	**1.0E-2**	-	-	**3.1E-2**	**1.6E-3**
***SERPINE1***	***Serpine1***	**0.021**	**0.018**	**0.02**	**0.129**	**1.6E-2**	**4.5E-5**	**9.6E-3**	**1.7E-2**	**3.3E-8**
*TNF*	*Tnf*	0.004	-0.003	0.007	0.051	2.3E-2	3.7E-1	2.6E-1	4.9E-2	4.5E-2
*TLE4*	*Tle4*	-0.004	-0.009	0.006	-0.07	2.3E-2	1.0E-3	3.1E-1	3.0E-1	1.0E-3
*SCGB1A1*	*Scgb1a1*	0.017	0.163	0.09	0.736	3.8E-2	1.2E-3	5.1E-2	1.8E-2	4.6E-6

* The beta coefficient corresponds to the mean change in gene expression per day during the studied period.

† Adjusted p-value (B-H method). ‡ Combined p-value for all data sets (human and murine) using the weighted z-score method.

Empty boxes (-) indicate that the gene was not represented on the chip. Bolded rows indicate genes with at least one significant probe per gene in all tested data sets (adjusted p-value <0.05).

In order to disentangle pre- and postnatal expression patterns in the murine data sets, separate pre- and postnatal analyses were attempted. However, this subgroup analysis was not meaningful for the SW and C56BL6 data sets because of substantially reduced sample size. The A/J data contains two prenatal time points (day 11 and 17), each with 4 unique samples and Table E6 shows overlapping results for human and prenatal A/J data. Eight of the previously identified 19 genes with consistent expression pattern across human and murine data sets (Table 4) were also identified when prenatal A/J data was used (including *Edn1*).

## Discussion

Little is known about the role of most asthma susceptibility genes during human lung development. Here we present a thorough evaluation of gene expression patterns of current published asthma genes in the developing human and murine lung. While there was no general over-representation of asthma genes among differentially expressed genes, some asthma genes were consistently differentially expressed in multiple developing lung transcriptomes, e.g. *NOD1, EDN1, CCL5, RORA *and *HLA-G *suggesting key functional roles in lung development.

Determinants for a normal lung development are critical not only early in life, but also for later lung function. Longitudinal studies have shown that infants with reduced lung function have an increased risk of developing asthma and respiratory illness later in life [97,98]. Shared genetic factors for reduced lung function in children with asthma and adults who smoke (e.g. *MMP12 *variants) emphasize the role of genetics on long term lung function [99]. *Wnt *signaling genes (e.g. *Wif1, Wisp1*) were not identified as asthma genes in our literature search, and were thus not included in our analyses. In our previous article by Sharma et al, *Wif1 *and *Wisp1 *were differentially expressed during fetal lung development and polymorphisms in these genes also showed association with lung function measured as FEV1 and FVC, but association to asthma per se was not tested [14].

The transcriptional control of lung morphogenesis is key for normal development from primordium to a fully differentiated, functioning organ [100,101]. Human lung growth has historically been categorised into five stages based on histological and anatomical characteristics: embryonic (26 days to 5 weeks), pseudoglandular (5-16 weeks), canalicular (16-26 weeks), saccular (26 weeks to birth), and alveolar (birth to 6 months) [100]. Additional "molecular" phases within the pseudoglandular stage have been observed, which extends our knowledge of lung development beyond traditional embryology [15].

GWAS have contributed to important knowledge about underlying functional genetics in many complex diseases [102]. The majority of trait associated SNPs show weak to moderate effect sizes, which supports earlier evidence that complex diseases result from several genetic and, often, environmental factors. Evidence of a functional role is also lacking for most identified genes. In order to increase our understanding of the mechanism and potential function of asthma susceptibility genes identified in published GWAS and "classic" asthma candidate genes, we evaluated their gene expression patterns in the developing human lung. Comparative analyses also showed that many of the differentially expressed genes in the human data set were also differentially expressed during murine lung development. Among the GWAS asthma genes, *ROBO1, RORA, HLA-DQB1, IL2RB *and *PDE10A *were differentially expressed in the human data. These genes represent a wide range of structural and ontological families with different assumed functions, but their potential involvement in lung development has previously not been thoroughly evaluated. Regulation of cytokine production and cell activation were the most significant bio-ontologic attributes to genes differentialy expressed during lung development.

Using the murine data sets for comparative analyses, *RORA*, which encodes for a nuclear hormone receptor, showed the most consistent expression pattern (expression positively correlated with gestational age in all data sets). *ROBO1 *expression was on the other hand negatively correlated with gestational age in all tested data sets (albeit significant in only 2/3 sets), which indicates an important effect early in the developing lung and then a diminishing effect over time. The ROBO1 protein is involved in axon guidance and neuronal precursor cell migration. *PTGDR, WDR36, PRNP, DENND1B, PDE4D, TLE4 *and *TSLP *also showed weak evidence of differential expression in the human data using adjusted p < 0.05 as cut off (Additional file  1, Table E2), but none showed consistent gene expression patterns in the murine data sets.

*NOD1 *showed the strongest evidence for differential expression in the human data and this pattern was consistent in the C57BL6 strain. However, *Nod1 *was not represented on the platforms used for analyses on the A/J and SW strains and could thus not be evaluated in these data sets (also true for another asthma gene with consistent expression patterns, *PCDH1 *[52]). *NOD1 *encodes for a cytosolic protein which contains an N-terminal caspase recruitment domain (CARD) and plays an important role for recognition of bacterial compounds and initiation of the innate immune response [103]. Little is known about the role of *NOD1 *during lung development and our findings indicate that *NOD1 *could have important contribution.

*EDN1 *was the second most differentially expressed asthma gene in the human data set and very consistent expression patterns were found in all murine data sets. Also for the embryonic stage analyses (pseudoglandular vs canalicular), *EDN1 *was among the most highly differentially expressed genes. In general, embryonic stage results were very similar to the results using time as a continuous variable. EDN1 belong to a family of secreted peptides produced by vascular endothelial cells with multiple effects on cardiovascular, neural, pulmonary and renal physiology [104,105]. EDN1 shows involvement in pulmonary hypertension, fibrosis, obstructive diseases and acute lung injury, and is also required for the normal development of several tissues. Mice lacking the *Edn1 *gene die of respiratory failure at birth and show severe craniofacial abnormalities, as well as cardiovascular defects [106,107]. Transgenic mice with lung-specific over-expression of the human *EDN1 *gene develop, on the other hand, chronic lung inflammation and fibrosis [108]. *Edn1 *heterozygous knockout mice also show increased bronchial responsiveness and these result link *EDN1 *functionally to asthma and obstructive diseases [72]. To date, three studies report significant association between *EDN1 *and asthma [41,109,110]. Our data, as well as previous studies, point to an important role for *EDN1 *in normal lung development, which warrants further studies.

Our study has several limitations. Our 38 human lung tissue samples were restricted to the pseudoglandular and canalicular stages. Information about key exposures that could influence gene expression patterns, such as maternal smoking, residential area, and parental allergy is not available. Thirty-eight samples are a relatively small sample size for expression analyses due to human biological variation and fetal lung tissue during the later stages of gestation was not available. It is possible that some asthma genes are important for human lung development during the later stages of gestation, but we were not able to evaluate this with our current data set. To complement the human data, we analysed expression patterns from early gestational to postnatal stages of lung development in three different murine strains. We used this murine data to replicate, in silico, the human results in the early stages and to infer human gene expression pattern in the later stages of the developing lung. Also, the microarray platforms used in the included data sets do not entirely cover the human (and murine) transcriptome and important genes may have been missed (e.g. *GPRA/NPSR1 *[111] is not represented on the U133 Plus 2.0 microarray chip and could not be evaluated). Protein analyses could provide a better view to understand specific gene functions and the post-transcriptional regulation level, but such data was not available in our study. Our asthma gene list represents genes that met our predefined criteria for asthma association, and some genes genes may have been missed (e.g. those only captured by the search terms "family based study" AND "asthma"). Given the rapid rate at which novel asthma susceptibility loci are being discovered, some of the most recent asthma genes may have been missed. These may introduce a potential null bias in the analysis.

## Conclusions

We have evaluated gene expression patterns of asthma susceptibility genes identified via a comprehensive literature search of candidate gene studies and GWAS published to date. We found strong and consistent evidence of differential expression of several asthma genes in the developing human and murine lung. Among genes identified in asthma GWAS, *ROBO1, RORA, HLA-DQB1, IL2RB *and *PDE10A *showed most consistent expression patterns and from asthma candidate genes, e.g. *NOD1, EDN1, CCL5 *and *HLA-G *were identified. Our analyses provide functional insight about asthma susceptibility genes during normal lung development, which improves our understanding about normal and pathological processes related to respiratory diseases in children and adults.

## List of abbreviations

CI: Confidence interval; DAVID: Database for Annotation, Visualization and Integrated Discovery; GEO: Gene Expression Omnibus; GO: Gene ontology; GWAS: Genome wide association studies; NCBI: National Center for Biotechnology Information; OR: Odds ratio; qPCR: Quantitative real time polymerase chain reaction.

## Competing interests

All authors declare no competing interests and no support from any organisation for the submitted work; no financial relationships with any organisations that might have an interest in the submitted work in the previous 3 years; no other relationships or activities that could appear to have influenced the submitted work.

## Authors' contributions

EM carried out the literature search and the statistical analyses together with ATK, SS and VJC. EM, RG, JSL, TJM, STW and KGT participated in the design and planning of the study. EM, ATK and KGT drafted the manuscript. All authors read and approved the final manuscript.

## Supplementary Material

Additional file 1 **Supplementary Tables and Figure**. Expression analysis of asthma candidate genes during human and murine lung development. Click here for file

## References

[B1] Ober C Hoffjan S Asthma genetics 2006: the long and winding road to gene discovery Genes Immun 2006 7 2 95 100 10.1038/sj.gene.6364284 16395390

[B2] Rogers AJ Raby BA Lasky-Su JA Murphy A Lazarus R Klanderman BJ Sylvia JS Ziniti JP Lange C Celedon JC Silverman EK Weiss ST Assessing the reproducibility of asthma candidate gene associations, using genome-wide data Am J Respir Crit Care Med 2009 179 12 1084 1090 10.1164/rccm.200812-1860OC 19264973 PMC2695495

[B3] Gudbjartsson DF Bjornsdottir US Halapi E Helgadottir A Sulem P Jonsdottir GM Thorleifsson G Helgadottir H Steinthorsdottir V Stefansson H Williams C Hui J Beilby J Warrington NM James A Palmer LJ Koppelman GH Heinzmann A Krueger M Boezen HM Wheatley A Altmuller J Shin HD Uh ST Cheong HS Jonsdottir B Gislason D Park CS Rasmussen LM Porsbjerg C Sequence variants affecting eosinophil numbers associate with asthma and myocardial infarction Nat Genet 2009 41 3 342 347 10.1038/ng.323 19198610

[B4] Hancock DB Romieu I Shi M Sienra-Monge JJ Wu H Chiu GY Li H del Rio-Navarro BE Willis-Owens SA Weiss ST Raby BA Gao H Eng C Chapela R Burchard EG Tang H Sullivan PF London SJ Genome-wide association study implicates chromosome 9q21.31 as a susceptibility locus for asthma in mexican children PLoS Genet 2009 5 8 e1000623 10.1371/journal.pgen.1000623 19714205 PMC2722731

[B5] Himes BE Hunninghake GM Baurley JW Rafaels NM Sleiman P Strachan DP Wilk JB Willis-Owen SA Klanderman B Lasky-Su J Lazarus R Murphy AJ Soto-Quiros ME Avila L Beaty T Mathias RA Ruczinski I Barnes KC Celedon JC Cookson WO Gauderman WJ Gilliland FD Hakonarson H Lange C Moffatt MF O'Connor GT Raby BA Silverman EK Weiss ST Genome-wide association analysis identifies PDE4D as an asthma-susceptibility gene Am J Hum Genet 2009 84 5 581 593 10.1016/j.ajhg.2009.04.006 19426955 PMC2681010

[B6] Li X Howard TD Zheng SL Haselkorn T Peters SP Meyers DA Bleecker ER Genome-wide association study of asthma identifies RAD50-IL13 and HLA-DR/DQ regions J Allergy Clin Immunol 2010 125 2 328 335 e311 10.1016/j.jaci.2009.11.018 20159242 PMC2824608

[B7] Mathias RA Grant AV Rafaels N Hand T Gao L Vergara C Tsai YJ Yang M Campbell M Foster C Gao P Togias A Hansel NN Diette G Adkinson NF Liu MC Faruque M Dunston GM Watson HR Bracken MB Hoh J Maul P Maul T Jedlicka AE Murray T Hetmanski JB Ashworth R Ongaco CM Hetrick KN Doheny KF A genome-wide association study on African-ancestry populations for asthma J Allergy Clin Immunol 2009 10.1016/j.jaci.2009.08.031 PMC3606015 19910028

[B8] Moffatt MF Gut IG Demenais F Strachan DP Bouzigon E Heath S von Mutius E Farrall M Lathrop M Cookson WO consortium G A large-scale, consortium-based genomewide association study of asthma N Engl J Med 2010 363 13 1211 1221 10.1056/NEJMoa0906312 20860503 PMC4260321

[B9] Moffatt MF Kabesch M Liang L Dixon AL Strachan D Heath S Depner M von Berg A Bufe A Rietschel E Heinzmann A Simma B Frischer T Willis-Owen SA Wong KC Illig T Vogelberg C Weiland SK von Mutius E Abecasis GR Farrall M Gut IG Lathrop GM Cookson WO Genetic variants regulating ORMDL3 expression contribute to the risk of childhood asthma Nature 2007 448 7152 470 473 10.1038/nature06014 17611496

[B10] Sleiman PM Flory J Imielinski M Bradfield JP Annaiah K Willis-Owen SA Wang K Rafaels NM Michel S Bonnelykke K Zhang H Kim CE Frackelton EC Glessner JT Hou C Otieno FG Santa E Thomas K Smith RM Glaberson WR Garris M Chiavacci RM Beaty TH Ruczinski I Orange JM Allen J Spergel JM Grundmeier R Mathias RA Christie JD Variants of DENND1B associated with asthma in children N Engl J Med 2010 362 1 36 44 10.1056/NEJMoa0901867 20032318

[B11] Barker DJ Martyn CN The maternal and fetal origins of cardiovascular disease J Epidemiol Community Health 1992 46 1 8 11 10.1136/jech.46.1.8 1573367 PMC1059485

[B12] Barker DJ In utero programming of chronic disease Clin Sci (Lond) 1998 95 2 115 128 10.1042/CS19980019 9680492

[B13] Stick S Pediatric origins of adult lung disease. 1. The contribution of airway development to paediatric and adult lung disease Thorax 2000 55 7 587 594 10.1136/thorax.55.7.587 10856320 PMC1745803

[B14] Sharma S Tantisira K Carey V Murphy AJ Lasky-Su J Celedon JC Lazarus R Klanderman B Rogers A Soto-Quiros M Avila L Mariani T Gaedigk R Leeder S Torday J Warburton D Raby B Weiss ST A Role for WNT-Signaling Genes in the Pathogenesis of Impaired Lung Function in Asthma Am J Respir Crit Care Med 2009 10.1164/rccm.200907-1009OC PMC2822972 19926868

[B15] Kho AT Bhattacharya S Tantisira KG Carey VJ Gaedigk R Leeder JS Kohane IS Weiss ST Mariani TJ Transcriptomic analysis of human lung development Am J Respir Crit Care Med 2009 181 1 54 63 19815808 10.1164/rccm.200907-1063OC PMC2797628

[B16] Bonner AE Lemon WJ You M Gene expression signatures identify novel regulatory pathways during murine lung development: implications for lung tumorigenesis J Med Genet 2003 40 6 408 417 10.1136/jmg.40.6.408 12807961 PMC1735509

[B17] Mariani TJ Reed JJ Shapiro SD Expression profiling of the developing mouse lung: insights into the establishment of the extracellular matrix Am J Respir Cell Mol Biol 2002 26 5 541 548 11970905 10.1165/ajrcmb.26.5.2001-00080c

[B18] Naxerova K Bult CJ Peaston A Fancher K Knowles BB Kasif S Kohane IS Analysis of gene expression in a developmental context emphasizes distinct biological leitmotifs in human cancers Genome Biol 2008 9 7 R108 10.1186/gb-2008-9-7-r108 18611264 PMC2530866

[B19] Weiss ST Raby BA Rogers A Asthma genetics and genomics 2009 Curr Opin Genet Dev 2009 19 3 279 282 10.1016/j.gde.2009.05.001 19481925

[B20] Vercelli D Discovering susceptibility genes for asthma and allergy Nat Rev Immunol 2008 8 3 169 182 10.1038/nri2257 18301422

[B21] Benjamini Y Hochberg Y Controlling the false discovery rate: a practical and powerful approach to multiple testing J R Stat Soc B 1995 57 289 300

[B22] Dennis G Jr Sherman BT Hosack DA Yang J Gao W Lane HC Lempicki RA DAVID: Database for Annotation, Visualization, and Integrated Discovery Genome Biol 2003 4 5 P3 10.1186/gb-2003-4-5-p3 12734009 PMC193660

[B23] Huang da W Sherman BT Lempicki RA Systematic and integrative analysis of large gene lists using DAVID bioinformatics resources Nat Protoc 2009 4 1 44 57 19131956 10.1038/nprot.2008.211

[B24] Bailey MT Kierstein S Sharma S Spaits M Kinsey SG Tliba O Amrani Y Sheridan JF Panettieri RA Haczku A Social stress enhances allergen-induced airway inflammation in mice and inhibits corticosteroid responsiveness of cytokine production J Immunol 2009 182 12 7888 7896 10.4049/jimmunol.0800891 19494313 PMC2767120

[B25] Barton SJ Koppelman GH Vonk JM Browning CA Nolte IM Stewart CE Bainbridge S Mutch S Rose-Zerilli MJ Postma DS Maniatis N Henry AP Hall IP Holgate ST Tighe P Holloway JW Sayers I PLAUR polymorphisms are associated with asthma, PLAUR levels, and lung function decline J Allergy Clin Immunol 2009 123 6 1391 1400 e1317 10.1016/j.jaci.2009.03.014 19443020

[B26] Batra J Pratap Singh T Mabalirajan U Sinha A Prasad R Ghosh B Association of inducible nitric oxide synthase with asthma severity, total serum immunoglobulin E and blood eosinophil levels Thorax 2007 62 1 16 22 10.1136/thx.2006.057935 17189532 PMC2111289

[B27] Berlin AA Lincoln P Tomkinson A Lukacs NW Inhibition of stem cell factor reduces pulmonary cytokine levels during allergic airway responses Clin Exp Immunol 2004 136 1 15 20 10.1111/j.1365-2249.2004.02404.x 15030509 PMC1809010

[B28] Berry MA Hargadon B Shelley M Parker D Shaw DE Green RH Bradding P Brightling CE Wardlaw AJ Pavord ID Evidence of a role of tumor necrosis factor alpha in refractory asthma N Engl J Med 2006 354 7 697 708 10.1056/NEJMoa050580 16481637

[B29] Bierbaum S Nickel R Koch A Lau S Deichmann KA Wahn U Superti-Furga A Heinzmann A Polymorphisms and haplotypes of acid mammalian chitinase are associated with bronchial asthma Am J Respir Crit Care Med 2005 172 12 1505 1509 10.1164/rccm.200506-890OC 16179638 PMC2718453

[B30] Bosse Y Lemire M Poon AH Daley D He JQ Sandford A White JH James AL Musk AW Palmer LJ Raby BA Weiss ST Kozyrskyj AL Becker A Hudson TJ Laprise C Asthma and genes encoding components of the vitamin D pathway Respir Res 2009 10 98 10.1186/1465-9921-10-98 19852851 PMC2779188

[B31] Buckova D Izakovicova Holla L Vacha J Polymorphism 4G/5G in the plasminogen activator inhibitor-1 (PAI-1) gene is associated with IgE-mediated allergic diseases and asthma in the Czech population Allergy 2002 57 5 446 448 10.1034/j.1398-9995.2002.03582.x 11972486

[B32] Cameron L Depner M Kormann M Klopp N Illig T von Mutius E Kabesch M Genetic variation in CRTh2 influences development of allergic phenotypes Allergy 2009 64 10 1478 1485 10.1111/j.1398-9995.2009.02053.x 19392992

[B33] Castro-Giner F Kunzli N Jacquemin B Forsberg B de Cid R Sunyer J Jarvis D Briggs D Vienneau D Norback D Gonzalez JR Guerra S Janson C Anto JM Wjst M Heinrich J Estivill X Kogevinas M Traffic-related air pollution, oxidative stress genes, and asthma (ECHRS) Environ Health Perspect 2009 117 12 1919 1924 20049212 10.1289/ehp.0900589 PMC2799467

[B34] Chatila TA Interleukin-4 receptor signaling pathways in asthma pathogenesis Trends Mol Med 2004 10 10 493 499 10.1016/j.molmed.2004.08.004 15464449

[B35] Chatterjee R Batra J Das S Sharma SK Ghosh B Genetic association of acidic mammalian chitinase with atopic asthma and serum total IgE levels J Allergy Clin Immunol 2008 122 1 202 208 208 e201-207 10.1016/j.jaci.2008.04.030 18602573

[B36] Chelbi H Ghadiri A Lacheb J Ghandil P Hamzaoui K Hamzaoui A Combadiere C A polymorphism in the CCL2 chemokine gene is associated with asthma risk: a case-control and a family study in Tunisia Genes Immun 2008 9 7 575 581 10.1038/gene.2008.50 18615095

[B37] Chen YQ Shi HZ CD28/CTLA-4--CD80/CD86 and ICOS--B7RP-1 costimulatory pathway in bronchial asthma Allergy 2006 61 1 15 26 10.1111/j.1398-9995.2006.01008.x 16364152

[B38] Cho SH Hall IP Wheatley A Dewar J Abraha D Del Mundo J Lee H Oh CK Possible role of the 4G/5G polymorphism of the plasminogen activator inhibitor 1 gene in the development of asthma J Allergy Clin Immunol 2001 108 2 212 214 10.1067/mai.2001.117260 11496236

[B39] Chu EK Cheng J Foley JS Mecham BH Owen CA Haley KJ Mariani TJ Kohane IS Tschumperlin DJ Drazen JM Induction of the plasminogen activator system by mechanical stimulation of human bronchial epithelial cells Am J Respir Cell Mol Biol 2006 35 6 628 638 10.1165/rcmb.2006-0040OC 16794260 PMC2643292

[B40] Crosby JR Guha M Tung D Miller DA Bender B Condon TP York-DeFalco C Geary RS Monia BP Karras JG Gregory SA Inhaled CD86 antisense oligonucleotide suppresses pulmonary inflammation and airway hyper-responsiveness in allergic mice J Pharmacol Exp Ther 2007 321 3 938 946 10.1124/jpet.106.119214 17389243

[B41] Daley D Lemire M Akhabir L Chan-Yeung M He JQ McDonald T Sandford A Stefanowicz D Tripp B Zamar D Bosse Y Ferretti V Montpetit A Tessier MC Becker A Kozyrskyj AL Beilby J McCaskie PA Musk B Warrington N James A Laprise C Palmer LJ Pare PD Hudson TJ Analyses of associations with asthma in four asthma population samples from Canada and Australia Hum Genet 2009 125 4 445 459 10.1007/s00439-009-0643-8 19247692

[B42] Goenka S Kaplan MH Transcriptional regulation by STAT6 Immunol Res 2011 50 1 87 96 10.1007/s12026-011-8205-2 21442426 PMC3107597

[B43] Hattori T Konno S Hizawa N Isada A Takahashi A Shimizu K Gao P Beaty TH Barnes KC Huang SK Nishimura M Genetic variants in the mannose receptor gene (MRC1) are associated with asthma in two independent populations Immunogenetics 2009 61 11-12 731 738 10.1007/s00251-009-0403-x 19902202

[B44] Higa S Hirano T Mayumi M Hiraoka M Ohshima Y Nambu M Yamaguchi E Hizawa N Kondo N Matsui E Katada Y Miyatake A Kawase I Tanaka T Association between interleukin-18 gene polymorphism 105A/C and asthma Clin Exp Allergy 2003 33 8 1097 1102 10.1046/j.1365-2222.2003.01739.x 12911784

[B45] Hong X Zhou H Tsai HJ Wang X Liu X Wang B Xu X Xu X Cysteinyl leukotriene receptor 1 gene variation and risk of asthma Eur Respir J 2009 33 1 42 48 10.1183/09031936.00057708 18829683

[B46] Hossny EM Amr NH Elsayed SB Nasr RA Ibraheim EM Association of polymorphisms in the mast cell chymase gene promoter region (-1903 g/A) and (TG)n(GA)m repeat downstream of the gene with bronchial asthma in children J Investig Allergol Clin Immunol 2008 18 5 376 381 18973102

[B47] Imada Y Fujimoto M Hirata K Hirota T Suzuki Y Saito H Matsumoto K Akazawa A Katsunuma T Yoshihara S Ebisawa M Shibasaki M Arinami T Tamari M Noguchi E Large scale genotyping study for asthma in the Japanese population BMC Res Notes 2009 2 54 10.1186/1756-0500-2-54 19335888 PMC2674055

[B48] Imboden M Nicod L Nieters A Glaus E Matyas G Bircher AJ Ackermann-Liebrich U Berger W Probst-Hensch NM The common G-allele of interleukin-18 single-nucleotide polymorphism is a genetic risk factor for atopic asthma. The SAPALDIA Cohort Study Clin Exp Allergy 2006 36 2 211 218 10.1111/j.1365-2222.2006.02424.x 16433859

[B49] Islam T Breton C Salam MT McConnell R Wenten M Gauderman WJ Conti D Van Den Berg D Peters JM Gilliland FD Role of inducible nitric oxide synthase in asthma risk and lung function growth during adolescence Thorax 2009 65 2 139 145 19996333 10.1136/thx.2009.114355

[B50] Jaradat M Stapleton C Tilley SL Dixon D Erikson CJ McCaskill JG Kang HS Angers M Liao G Collins J Grissom S Jetten AM Modulatory role for retinoid-related orphan receptor alpha in allergen-induced lung inflammation Am J Respir Crit Care Med 2006 174 12 1299 1309 10.1164/rccm.200510-1672OC 16973978 PMC2648295

[B51] Jetten AM Retinoid-related orphan receptors (RORs): critical roles in development, immunity, circadian rhythm, and cellular metabolism Nucl Recept Signal 2009 7 e003 19381306 10.1621/nrs.07003 PMC2670432

[B52] Koppelman GH Meyers DA Howard TD Zheng SL Hawkins GA Ampleford EJ Xu J Koning H Bruinenberg M Nolte IM van Diemen CC Boezen HM Timens W Whittaker PA Stine OC Barton SJ Holloway JW Holgate ST Graves PE Martinez FD van Oosterhout AJ Bleecker ER Postma DS Identification of PCDH1 as a novel susceptibility gene for bronchial hyperresponsiveness Am J Respir Crit Care Med 2009 180 10 929 935 10.1164/rccm.200810-1621OC 19729670 PMC2778155

[B53] Kowal K Bodzenta-Lukaszyk A Pampuch A Szmitkowski M Zukowski S Donati MB Iacoviello L Analysis of -675 4 g/5 G SERPINE1 and C-159T CD14 polymorphisms in house dust mite-allergic asthma patients J Investig Allergol Clin Immunol 2008 18 4 284 292 18714537

[B54] Kowal K Moniuszko M Zukowski S Bodzenta-Lukaszyk A Concentrations of plasminogen activator inhibitor-1 (PAI-1) and urokinase plasminogen activator (uPA) in induced sputum of asthma patients after allergen challenge Folia Histochem Cytobiol 2010 48 4 518 523 10.2478/v10042-010-0075-2 21478092

[B55] Kucharewicz I Mogielnicki A Kasacka I Buczko W Bodzenta-Lukaszyk A Plasmin system regulation in an ovalbumin-induced rat model of asthma Int Arch Allergy Immunol 2008 147 3 190 196 10.1159/000142041 18594148

[B56] Lee CC Lin WY Wan L Tsai Y Tsai CH Huang CM Chen CP Tsai FJ Association of interleukin-18 gene polymorphism with asthma in Chinese patients J Clin Lab Anal 2008 22 1 39 44 10.1002/jcla.20218 18200581 PMC6649026

[B57] Lee JH Moore JH Park SW Jang AS Uh ST Kim YH Park CS Park BL Shin HD Genetic interactions model among Eotaxin gene polymorphisms in asthma J Hum Genet 2008 53 10 867 875 10.1007/s10038-008-0314-y 18712274

[B58] Li H Romieu I Sienra-Monge JJ Ramirez-Aguilar M Estela Del Rio-Navarro B Kistner EO Gjessing HK Lara-Sanchez Idel C Chiu GY London SJ Genetic polymorphisms in arginase I and II and childhood asthma and atopy J Allergy Clin Immunol 2006 117 1 119 126 10.1016/j.jaci.2005.09.026 16387594 PMC1450009

[B59] Li YF Tseng PJ Lin CC Hung CL Lin SC Su WC Huang YL Sung FC Tai CK NAD(P)H: Quinone oxidoreductase 1, glutathione S-transferase M1, environmental tobacco smoke exposure, and childhood asthma Mutat Res 2009 678 1 53 58 19591959 10.1016/j.mrgentox.2009.06.008

[B60] Liu Q Xia Y Zhang W Li J Wang P Li H Wei C Gong Y A functional polymorphism in the SPINK5 gene is associated with asthma in a Chinese Han Population BMC Med Genet 2009 10 59 19534795 10.1186/1471-2350-10-59 PMC2709655

[B61] Madore AM Perron S Turmel V Laviolette M Bissonnette EY Laprise C Alveolar macrophages in allergic asthma: an expression signature characterized by heat shock protein pathways Hum Immunol 2010 71 2 144 150 10.1016/j.humimm.2009.11.005 19913588 PMC7124256

[B62] Martinez B Barrios K Vergara C Mercado D Jimenez S Gusmao L Caraballo L A NOS1 gene polymorphism associated with asthma and specific immunoglobulin E response to mite allergens in a Colombian population Int Arch Allergy Immunol 2007 144 2 105 113 10.1159/000103221 17536218

[B63] Masumoto J Yang K Varambally S Hasegawa M Tomlins SA Qiu S Fujimoto Y Kawasaki A Foster SJ Horie Y Mak TW Nunez G Chinnaiyan AM Fukase K Inohara N Nod1 acts as an intracellular receptor to stimulate chemokine production and neutrophil recruitment in vivo J Exp Med 2006 203 1 203 213 10.1084/jem.20051229 16418393 PMC2118074

[B64] Matsuzaki S Ishizuka T Hisada T Aoki H Komachi M Ichimonji I Utsugi M Ono A Koga Y Dobashi K Kurose H Tomura H Mori M Okajima F Lysophosphatidic acid inhibits CC chemokine ligand 5/RANTES production by blocking IRF-1-mediated gene transcription in human bronchial epithelial cells J Immunol 2010 185 8 4863 4872 10.4049/jimmunol.1000904 20861350

[B65] McKay A Komai-Koma M MacLeod KJ Campbell CC Kitson SM Chaudhuri R Thomson L McSharry C Liew FY Thomson NC Interleukin-18 levels in induced sputum are reduced in asthmatic and normal smokers Clin Exp Allergy 2004 34 6 904 910 10.1111/j.1365-2222.2004.01973.x 15196278

[B66] Millstein JCD Gilliland FD Gauderman WJ A testing framework for identifying suseptibility genes in the presence of epistasis Am J Hum Genet 2006 78 15 27 10.1086/498850 16385446 PMC1380213

[B67] Minelli C Granell R Newson R Rose-Zerilli MJ Torrent M Ring SM Holloway JW Shaheen SO Henderson JA Glutathione-S-transferase genes and asthma phenotypes: a Human Genome Epidemiology (HuGE) systematic review and meta-analysis including unpublished data Int J Epidemiol 2010 39 2 539 562 10.1093/ije/dyp337 20032267 PMC2846443

[B68] Moller-Larsen S Nyegaard M Haagerup A Vestbo J Kruse TA Borglum AD Association analysis identifies TLR7 and TLR8 as novel risk genes in asthma and related disorders Thorax 2008 63 12 1064 1069 10.1136/thx.2007.094128 18682521

[B69] Movahedi M Moin M Gharagozlou M Aghamohammadi A Dianat S Moradi B Nicknam MH Nikbin B Amirzargar A Association of HLA class II alleles with childhood asthma and Total IgE levels Iran J Allergy Asthma Immunol 2008 7 4 215 220 19052351

[B70] Munthe-Kaas MC Carlsen KH Haland G Devulapalli CS Gervin K Egeland T Carlsen KL Undlien D T cell-specific T-box transcription factor haplotype is associated with allergic asthma in children J Allergy Clin Immunol 2008 121 1 51 56 10.1016/j.jaci.2007.07.068 17949803

[B71] Munthe-Kaas MC Carlsen KL Carlsen KH Egeland T Haland G Devulapalli CS Akselsen H Undlien D HLA Dr-Dq haplotypes and the TNFA-308 polymorphism: associations with asthma and allergy Allergy 2007 62 9 991 998 10.1111/j.1398-9995.2007.01377.x 17686102

[B72] Nagase T Kurihara H Kurihara Y Aoki-Nagase T Nagai R Ouchi Y Disruption of ET-1 gene enhances pulmonary responses to methacholine via functional mechanism in knockout mice J Appl Physiol 1999 87 6 2020 2024 10601144 10.1152/jappl.1999.87.6.2020

[B73] Nicolae D Cox NJ Lester LA Schneider D Tan Z Billstrand C Kuldanek S Donfack J Kogut P Patel NM Goodenbour J Howard T Wolf R Koppelman GH White SR Parry R Postma DS Meyers D Bleecker ER Hunt JS Solway J Ober C Fine mapping and positional candidate studies identify HLA-G as an asthma susceptibility gene on chromosome 6p21 Am J Hum Genet 2005 76 2 349 357 10.1086/427763 15611928 PMC1196380

[B74] Pampuch A Kowal K Bodzenta-Lukaszyk A Di Castelnuovo A Chyczewski L Donati MB Iacoviello L The -675 4G/5G plasminogen activator inhibitor-1 promoter polymorphism in house dust mite-sensitive allergic asthma patients Allergy 2006 61 2 234 238 10.1111/j.1398-9995.2005.00948.x 16409202

[B75] Pegorier S Arouche N Dombret MC Aubier M Pretolani M Augmented epithelial endothelin-1 expression in refractory asthma J Allergy Clin Immunol 2007 120 6 1301 1307 10.1016/j.jaci.2007.09.023 17996929

[B76] Raby BA Hwang ES Van Steen K Tantisira K Peng S Litonjua A Lazarus R Giallourakis C Rioux JD Sparrow D Silverman EK Glimcher LH Weiss ST T-bet polymorphisms are associated with asthma and airway hyperresponsiveness Am J Respir Crit Care Med 2006 173 1 64 70 10.1164/rccm.200503-505OC 16179640 PMC2662983

[B77] Ray R Choi M Zhang Z Silverman GA Askew D Mukherjee AB Uteroglobin suppresses SCCA gene expression associated with allergic asthma J Biol Chem 2005 280 11 9761 9764 10.1074/jbc.C400581200 15677460

[B78] Saadi A Gao G Li H Wei C Gong Y Liu Q Association study between vitamin D receptor gene polymorphisms and asthma in the Chinese Han population: a case-control study BMC Med Genet 2009 10 71 19622139 10.1186/1471-2350-10-71 PMC2720948

[B79] Salam MT Islam T Gauderman WJ Gilliland FD Roles of arginase variants, atopy, and ozone in childhood asthma J Allergy Clin Immunol 2009 123 3 596 602 602 e591-598 10.1016/j.jaci.2008.12.020 19281908 PMC2913574

[B80] Sanz C Isidro-Garcia M Davila I Moreno E Laffond E Lorente F Analysis of 927T > C CYSLTRI and -444A > C LTC4S polymorphisms in patients with asthma J Investig Allergol Clin Immunol 2006 16 6 331 337 17153879

[B81] Seibold MA Reese TA Choudhry S Salam MT Beckman K Eng C Atakilit A Meade K Lenoir M Watson HG Thyne S Kumar R Weiss KB Grammer LC Avila P Schleimer RP Fahy JV Rodriguez-Santana J Rodriguez-Cintron W Boot RG Sheppard D Gilliland FD Locksley RM Burchard EG Differential enzymatic activity of common haplotypic versions of the human acidic Mammalian chitinase protein J Biol Chem 2009 284 29 19650 19658 10.1074/jbc.M109.012443 19435888 PMC2740590

[B82] Suttner K Rosenstiel P Depner M Schedel M Pinto LA Ruether A Adamski J Klopp N Illig T Vogelberg C Schreiber S von Mutius E Kabesch M TBX21 gene variants increase childhood asthma risk in combination with HLX1 variants J Allergy Clin Immunol 2009 123 5 1062 1068 1068 e1061-1068 10.1016/j.jaci.2009.02.025 19362357

[B83] Szalai C Kozma GT Nagy A Bojszko A Krikovszky D Szabo T Falus A Polymorphism in the gene regulatory region of MCP-1 is associated with asthma susceptibility and severity J Allergy Clin Immunol 2001 108 3 375 381 10.1067/mai.2001.117930 11544456

[B84] Szczepankiewicz A Breborowicz A Skibinska M Wilkosc M Tomaszewska M Hauser J Association analysis of brain-derived neurotrophic factor gene polymorphisms in asthmatic children Pediatr Allergy Immunol 2007 18 4 293 297 10.1111/j.1399-3038.2007.00525.x 17584309

[B85] Szczepankiewicz A Rose-Zerilli MJ Barton SJ Holgate ST Holloway JW Association analysis of brain-derived neurotrophic factor gene polymorphisms in asthmatic families Int Arch Allergy Immunol 2009 149 4 343 349 10.1159/000205580 19295238

[B86] van den Oord RA Sheikh A Filaggrin gene defects and risk of developing allergic sensitisation and allergic disorders: systematic review and meta-analysis Bmj 2009 339 b2433 10.1136/bmj.b2433 19589816 PMC2714678

[B87] Wang J Xu Y Zhao H Sui H Liang H Jiang X Genetic variations in chemoattractant receptor expressed on Th2 cells (CRTH2) is associated with asthma susceptibility in Chinese children Mol Biol Rep 2009 36 6 1549 1553 10.1007/s11033-008-9349-6 18777142

[B88] Wang JY Shyur SD Wang WH Liou YH Lin CG Wu YJ Wu LS The polymorphisms of interleukin 17A (IL17A) gene and its association with pediatric asthma in Taiwanese population Allergy 2009 64 7 1056 1060 10.1111/j.1398-9995.2009.01950.x 19210369

[B89] Vergara C Tsai YJ Grant AV Rafaels N Gao L Hand T Stockton M Campbell M Mercado D Faruque M Dunston G Beaty TH Oliveira RR Ponte EV Cruz AA Carvalho E Araujo MI Watson H Schleimer RP Caraballo L Nickel RG Mathias RA Barnes KC Gene encoding Duffy antigen/receptor for chemokines is associated with asthma and IgE in three populations Am J Respir Crit Care Med 2008 178 10 1017 1022 10.1164/rccm.200801-182OC 18827265 PMC2582596

[B90] White JH Chiano M Wigglesworth M Geske R Riley J White N Hall S Zhu G Maurio F Savage T Anderson W Cordy J Ducceschi M Vestbo J Pillai SG Identification of a novel asthma susceptibility gene on chromosome 1qter and its functional evaluation Hum Mol Genet 2008 17 13 1890 1903 10.1093/hmg/ddn087 18344558

[B91] Yamagata S Tomita K Sato R Niwa A Higashino H Tohda Y Interleukin-18-deficient mice exhibit diminished chronic inflammation and airway remodelling in ovalbumin-induced asthma model Clin Exp Immunol 2008 154 3 295 304 10.1111/j.1365-2249.2008.03772.x 18826499 PMC2633229

[B92] Yang CJ Liu YK Liu CL Shen CN Kuo ML Su CC Tseng CP Yen TC Shen CR Inhibition of acidic mammalian chitinase by RNA interference suppresses ovalbumin-sensitized allergic asthma Hum Gene Ther 2009 20 12 1597 1606 10.1089/hum.2008.092 19548841

[B93] Ye Q Fujita M Ouchi H Inoshima I Maeyama T Kuwano K Horiuchi Y Hara N Nakanishi Y Serum CC-10 in inflammatory lung diseases Respiration 2004 71 5 505 510 10.1159/000080636 15467329

[B94] Zeilinger S Pinto LA Nockher WA Depner M Klopp N Illig T von Mutius E Renz H Kabesch M The effect of BDNF gene variants on asthma in German children Allergy 2009 64 12 1790 1794 10.1111/j.1398-9995.2009.02131.x 19895626

[B95] Zheng XQ Li CC Xu DP Lin A Bao WG Yang GS Yan WH Analysis of the plasma soluble human leukocyte antigen-G and interleukin-10 levels in childhood atopic asthma Hum Immunol 2010 71 10 982 987 10.1016/j.humimm.2010.06.018 20600443

[B96] Zhou H Hong X Jiang S Dong H Xu X Analyses of associations between three positionally cloned asthma candidate genes and asthma or asthma-related phenotypes in a Chinese population BMC Med Genet 2009 10 123 10.1186/1471-2350-10-123 19951440 PMC2799396

[B97] Haland G Carlsen KC Sandvik L Devulapalli CS Munthe-Kaas MC Pettersen M Carlsen KH Reduced lung function at birth and the risk of asthma at 10 years of age N Engl J Med 2006 355 16 1682 1689 10.1056/NEJMoa052885 17050892

[B98] Martinez FD Wright AL Taussig LM Holberg CJ Halonen M Morgan WJ Asthma and wheezing in the first six years of life. The Group Health Medical Associates N Engl J Med 1995 332 3 133 138 10.1056/NEJM199501193320301 7800004

[B99] Hunninghake GM Cho MH Tesfaigzi Y Soto-Quiros ME Avila L Lasky-Su J Stidley C Melen E Soderhall C Hallberg J Kull I Kere J Svartengren M Pershagen G Wickman M Lange C Demeo DL Hersh CP Klanderman BJ Raby BA Sparrow D Shapiro SD Silverman EK Litonjua AA Weiss ST Celedon JC MMP12, lung function, and COPD in high-risk populations N Engl J Med 2009 361 27 2599 2608 10.1056/NEJMoa0904006 20018959 PMC2904064

[B100] Maeda Y Dave V Whitsett JA Transcriptional control of lung morphogenesis Physiol Rev 2007 87 1 219 244 10.1152/physrev.00028.2006 17237346

[B101] Franzdottir SR Axelsson IT Arason AJ Baldursson O Gudjonsson T Magnusson MK Airway branching morphogenesis in three dimensional culture Respir Res 2010 11 162 10.1186/1465-9921-11-162 21108827 PMC3002372

[B102] Hindorff LA Sethupathy P Junkins HA Ramos EM Mehta JP Collins FS Manolio TA Potential etiologic and functional implications of genome-wide association loci for human diseases and traits Proc Natl Acad Sci USA 2009 106 23 9362 9367 10.1073/pnas.0903103106 19474294 PMC2687147

[B103] Ting JP Duncan JA Lei Y How the noninflammasome NLRs function in the innate immune system Science 2010 327 5963 286 290 10.1126/science.1184004 20075243 PMC3943909

[B104] Fagan KA McMurtry IF Rodman DM Role of endothelin-1 in lung disease Respir Res 2001 2 2 90 101 10.1186/rr44 11686871 PMC59574

[B105] Stow LR Jacobs ME Wingo CS Cain BD Endothelin-1 gene regulation FASEB J 2011 25 1 16 28 10.1096/fj.10-161612 20837776 PMC3005421

[B106] Kurihara Y Kurihara H Oda H Maemura K Nagai R Ishikawa T Yazaki Y Aortic arch malformations and ventricular septal defect in mice deficient in endothelin-1 J Clin Invest 1995 96 1 293 300 10.1172/JCI118033 7615798 PMC185200

[B107] Kurihara Y Kurihara H Suzuki H Kodama T Maemura K Nagai R Oda H Kuwaki T Cao WH Kamada N Elevated blood pressure and craniofacial abnormalities in mice deficient in endothelin-1 Nature 1994 368 6473 703 710 10.1038/368703a0 8152482

[B108] Hocher B Schwarz A Fagan KA Thone-Reineke C El-Hag K Kusserow H Elitok S Bauer C Neumayer HH Rodman DM Theuring F Pulmonary fibrosis and chronic lung inflammation in ET-1 transgenic mice Am J Respir Cell Mol Biol 2000 23 1 19 26 10873149 10.1165/ajrcmb.23.1.4030

[B109] Immervoll T Loesgen S Dutsch G Gohlke H Herbon N Klugbauer S Dempfle A Bickeboller H Becker-Follmann J Ruschendorf F Saar K Reis A Wichmann HE Wjst M Fine mapping and single nucleotide polymorphism association results of candidate genes for asthma and related phenotypes Hum Mutat 2001 18 4 327 336 10.1002/humu.1194 11668616

[B110] Zhu G Carlsen K Carlsen KH Lenney W Silverman M Whyte MK Hosking L Helms P Roses AD Hay DW Barnes MR Anderson WH Pillai SG Polymorphisms in the endothelin-1 (EDN1) are associated with asthma in two populations Genes Immun 2008 9 1 23 29 10.1038/sj.gene.6364441 17960156

[B111] Melen E Bruce S Doekes G Kabesch M Laitinen T Lauener R Lindgren CM Riedler J Scheynius A van Hage-Hamsten M Kere J Pershagen G Wickman M Nyberg F Haplotypes of G protein-coupled receptor 154 are associated with childhood allergy and asthma Am J Respir Crit Care Med 2005 171 10 1089 1095 10.1164/rccm.200410-1317OC 15710598

