# Supplementary material for: Expression analysis of asthma candidate genes during human and murine lung development
Source: Respir Res. 2011 Jun 23;12(1):86. doi: 10.1186/1465-9921-12-86 (PMC3141421; doi:10.1186/1465-9921-12-86)
Supplement: Additional file 1 — Supplementary Tables and Figure. Expression analysis of asthma candidate genes during human and murine lung development. [file 1465-9921-12-86-S1.DOC]

**Additional file 1**

**Expression analysis of asthma candidate genes during human and murine lung development**

Erik Melén, Alvin T Kho, Sunita Sharma, Roger Gaedigk, J Steven Leeder, Thomas J Mariani, Vincent J Carey, Scott T Weiss and Kelan G Tantisira

| **Table E1. Asthma candidate genes identified through literature search** | | | | | | |
| --- | --- | --- | --- | --- | --- | --- |
| **Gene Name**  **(Official symbol, alias)** | **Chr.pos.** | **N*** | **Race/Ethnicity** | **Age** | **Pub Year** | **Reference** |
| **Genes identified in GWAS through September 2010** | | | | | | |
| Adrenergic, alpha-1B-, receptor, ADRA1B | 5q23-32 | 498/500,  163 F + replication | African American, African Caribbean, Caucasian | Mixed | 2009 |  |
| Crumbs homolog 1, CRB1 | 1q31-q32.1 | 793/1988, 917/1546, 1667/2045 | Caucasian, African American | Children / Mixed | 2009 |  |
| DENN/MADD domain containing 1B, DENND1B | 1q31.3 | 793/1988, 917/1546, 1667/2045 | Caucasian, African American | Children / Mixed | 2009 |  |
| Dipeptidyl peptidase 10 (DPP10) | 2q12 | 498/500,  163 F + replication | African American, African Caribbean, Caucasian | Mixed | 2009 |  |
| Gasdermin A GSDMA* | 17q21 | 10,365/16,110 | Caucasian | Mixed | 2010 |  |
| Gasdermin B (GSDMB) alias GSDML | 17q12 | 994/1243 | Caucasian | Children | 2007 |  |
|  |  | 10,365/16,110 | Caucasian | Mixed | 2010 |  |
| Glucan (1,4-alpha-), branching enzyme 1, GBE1 | 3p12.3 | 793/1988, 917/1546 | Caucasian | Children / Mixed | 2009 |  |
| Major histocompatibility complex, class II, DQ beta 1 (HLA-DQB1) | 6p21.3 | 10,365/16,110 | Caucasian | Mixed | 2010 |  |
| Interleukin 2 receptor, beta (IL2RB) | 22q13 | 10,365/16,110 | Caucasian | Mixed | 2010 |  |
| Interleukin 1 receptor-like 1 / Interleukin 18 receptor 1 (IL1RL1 /IL18R1) | 2q12 | 2,227/38,349 + replication | Caucasian, Korean | Mixed | 2009 |  |
|  |  | 10,365/16,110 | Caucasian | Mixed | 2010 |  |
| Interleukin 13 (IL13) | 5q31 | 10,365/16,110 | Caucasian | Mixed | 2010 |  |
| Interleukin 33 (IL33) | 9q24 | 2,227/38,349 + replication | Caucasian, Korean | Mixed | 2009 |  |
|  |  | 10,365/16,110 | Caucasian | Mixed | 2010 |  |
| V-myb myeloblastosis viral oncogene homolog (MYB) | 6q23 | 2,227/38,349 + replication | Caucasian, Korean | Mixed | 2009 |  |
| ORM1-like protein 3 (ORMDL3) |  | 994/1243 | Caucasian | Children | 2007 |  |
|  |  | 10,365/16,110 | Caucasian | Mixed | 2010 |  |
| Phosphodiesterase 4D, cAMP-specific, PDE4D | 5q12 | 359/846 + replication | Caucasian, Hispanic | Children, Mixed | 2009 |  |
| Phosphodiesterase 10A, PDE10A | 6q26 | 793/1988, 917/1546 | Caucasian | Children / Mixed | 2009 |  |
| Prion protein, PRNP | 20p13 | 498/500,  163 F + replication | African American, African Caribbean, Caucasian | Mixed | 2009 |  |
| Protein tyrosine phosphatase, receptor type, D (PTPRD) | 9p23-p24.3 | 793/1988, 917/1546 | Caucasian | Children / Mixed | 2009 |  |
| RAD50 homolog (RAD50) | 5q31 | 473/1892 | Caucasian | Adult | 2010 |  |
| RAR-related orphan receptor A (RORA) | 15q22 | 10,365/16,110 | Caucasian | Mixed | 2010 |  |
| Roundabout, axon guidance receptor, homolog 1, ROBO1 | 3p12 | 793/1988, 917/1546 | Caucasian | Children / Mixed | 2009 |  |
| SMAD family member 3 (SMAD3) | 15q22 | 10,365/16,110 | Caucasian | Mixed | 2010 |  |
| Solute carrier family 22 (organic cation/carnitine transporter), member 5 (SLC22A5) | 5q31 | 10,365/16,110 | Caucasian | Mixed | 2010 |  |
| Transducin-like enhancer of split 4 (TLE4) | 9q21.31 | 492 F | Hispanic | 9.0 | 2009 |  |
|  |  | 177 F | Hispanic | Children | 2009 |  |
| Thymic stromal lymphopoietin (TSLP) | 5q22.1 | 2,227/38,349 + replication | Caucasian, Korean | Mixed | 2009 |  |
| WD repeat domain 36 (WDR36) | 5q22 | 2,227/38,349 + replication | Caucasian, Korean | Mixed | 2009 |  |
|  |  |  |  |  |  |  |
| *** GSDMA is not represented on the Affy133 plus chip and therefore not included in the analyses. | | | | | | |
|  |  |  |  |  |  |  |
|  |  |  |  |  |  |  |
| **Genes identified from non-GWAS studies published 07/01/08-12/31/09** | | | | | | |
| Arginase, type II (ARG2) | 14q24.1-q24.3 | 2946 (450 asthma) | Caucasian, Hispanic | Children | 2009 |  |
|  |  | 433 F | Hispanic | Children | 2006 |  |
| Brain-derived neurotrophic factor, BDNF | 11p13 | 3099 pop, 655/767 | Caucasian | Children | 2009 |  |
|  |  | 341 F | Caucasian | Mixed | 2009 |  |
|  |  | 56/109 | Caucasian | Children | 2007 |  |
| CD86 molecule, CD86 | 3q21 | 388 F + replication | Caucasian | Mixed | 2009 |  |
| Chemokine (C-C motif) ligand 26 (CCL26 / Eotaxin 3 | 7q11.23 | 300/300 | Korean | Mixed | 2008 |  |
| Chemokine (C-C motif) ligand 2 / Monocyte chemoattractant protein-1 (CCL2/ MCP-1) | 17q11.2-q12 | 121/226, 99 F | North African | Children | 2008 |  |
|  |  | 160/303 | Caucasian | Children | 2001 |  |
| Chitinase, acidic /Acidic mammalian chitinase CHIA / AMCase | 1p13.1-p21. | 264/176, 301 F, 399 F + replication | African American, Latino, Puerto Rican | Mixed | 2009 |  |
|  |  | 270/292, 150/101 | North India | Adults, children | 2008 |  |
|  |  | 322/270 | Caucasian | Mixed | 2005 |  |
| Cysteinyl leukotriene receptor 1 (CYSLTR1) | Xq13.2-q21.1 | 170/347, 202/332 | Chinese | Mixed | 2009 |  |
|  |  | 130/78 | Caucasian | Mixed | 2006 |  |
| Cytochrome P450, family 2, subfamily R, polypeptide 1, CYP2R1 | 11p15.2 | 388 F + replication | Caucasian | Mixed | 2009 |  |
| Cytochrome P450, family 24, subfamily A, polypeptide 1, CYP24A1 | 20q13 | 388 F + replication | Caucasian | Mixed | 2009 |  |
| Duffy blood group, chemokine receptor (DARC) | 1q21-q22 | 125 F, 167 F, 82 F | African Caribbean, South American | Mixed | 2008 |  |
| Filaggrin (FLG) | 1q21 | Multiple | Mixed | Mixed | 2009 |  |
| G protein-coupled receptor 44 / Chemoattractant receptor-homologous molecule expressed on Th2 cells (GPR44 / CRTh2) | 11q12-q13.3 | 4264 pop (350 asthma) | Caucasian | Children | 2009 |  |
|  |  | 264/163 | Chinese | Children | 2009 |  |
| Hippocalcin-like 1 (HPCAL1) | 2p25.1 | 288/1032, 216 F, 541/744 | Japanese | Children | 2009 |  |
| Interleukin 17A (IL17A) | 6p12 | 481/546, 729/202 | Taiwanese | Children | 2009 |  |
| Major histocompatibility complex, class I, G, HLA-G | 6p21.3 | 1183 pop (543 asthma) | Chinese | Mixed | 2009 |  |
|  |  | 138 F + replication | Caucasian, Hutteries | Mixed | 2005 |  |
| Mannose receptor, C type 1, MRC1 | 10p12 | 446/424 + replication | Japanese, African America | Adult / Mixed | 2009 |  |
| Nitric oxide synthase 2, inducible, NOS2 | 17q11.2-q12 | 1596 pop (incl 150 asthma) | Caucasian / Latino | Children | 2009 |  |
|  |  | 230 F | N India | Mixed | 2007 |  |
| Opsin 3 (OPN3) | 1q43 | 294 F, 442 F, 404 F | Caucasian | Mixed | 2008 |  |
| Peroxisomal biogenesis factor 19 (PEX19) | 1q23.2 | 288/1032, 216 F, 541/744 | Japanese | Children | 2009 |  |
| Protocadherin 1, PCDH1 | 5q32-33 | 200 F + replication | Caucasian | Children | 2009 |  |
| Quinone oxidoreductase 1 (NQO1) | 16q22.1 | 6226 pop (874 asthma) | Taiwanese | Children | 2009 |  |
|  |  | 2577 pop (327) | Caucasian | Adults | 2009 |  |
|  |  | 2089 pop (321 asthma), 445 (72 asthma) | Caucasian, Hispanic, Mixed | Children | 2006 |  |
| Plasma urokinase plasminogen activator receptor (PLAUR / UPAR) | 19q13 | 341 F, 200 F, 46 F | Caucasian | Mixed | 2009 |  |
| Serine peptidase inhibitor, Kazal type 5 (SPINK5) | 5q31-32 | 669/711 | Chinese | 41.3 | 2009 |  |
| Serpin peptidase inhibitor / plasminogen activator inhibitor, type I (SERPINE1 / PAI-1), *4G/5G in/del* | 7q21.3-q22 | *372/160* | Caucasian | Adults | 2008 |  |
|  |  | *127/89* | Caucasian | Adults | 2006 |  |
|  |  | *159/186* | Caucasian | Adults | 2002 |  |
|  |  | *51 F* | Caucasian | Mixed | 2001 |  |
| T-box 21 (TBX21 / T-BET) | 17q21.32 | 3099 pop, 369/1248 | Caucasian | Children | 2009 |  |
|  |  | 106/491 | Caucasian | Children | 2008 |  |
|  |  | 582 F, 517/519 | Caucasian | Children, adults | 2006 |  |
| Toll-like receptor 8 (TLR8) | Xp22 | 135 F (270 asthmatics), 100 F (157 asthmatics) | Caucasian | Children | 2008 |  |
| Vitamin D receptor (VDR) | 12q13.11 | 567/523 | Chinese | Mixed | 2009 |  |
|  |  | 545 F, 723 F, 306 F, 644/751 | Caucasian | Children, Mixed | 2009 |  |
|  |  |  |  |  |  |  |
| F = Family based study  Pop = Population based study |  |  |  |  |  |  |
|  | | | | | | |
| **Additional asthma candidate genes identified in Vercelli D. *Nat Rev Immunol* 2008;8:169-182 (not identified above).** | | | | | | |
| Glutathione-S transferase M1  (GSTM1), deletion | 1p13 | Multiple studies | Multiple | Mixed | 2009 |  |
|  |  | Multiple studies | Multiple | Mixed | 2006 |  |
| Major histocompatibility complex, class II, DR beta 1 (HLA-DRB1) | 6p21.3 | 112/? | Caucasian (Iran) | Children | 2008 |  |
|  |  | 959 pop (268 asthma) | Caucasian | Children | 2007 |  |
|  |  | Multiple studies | Multiple | Mixed | 2006 |  |
| Interleukin 18 (IL18) | 11q22 | 545 F, 723 F, 306 F, 644/751 | Caucasian | Children, Mixed | 2009 |  |
|  |  | 201/60 | Chinese | Mixed | 2008 |  |
|  |  | 5734 pop (530 asthma) | Caucasian | Adults | 2006 |  |
|  |  | 497/85 | Japanese | Mixed | 2003 |  |
| Signal transducer and activator of transcription 6 (STAT6), SNP / GTn | 12q13 | Multiple studies | Multiple | Mixed | 2006 |  |
| Nitric oxide synthase 1 (NOS1), SNP / CA-repeat /microsatellites | 12q24 | 167/166 | Hispanic | Mixed | 2007 |  |
|  |  | Multiple studies | Multiple | Mixed | 2006 |  |
| Chymase 1 (CMA1) | 14q11 | 15/15 | North African | Children | 2008 |  |
|  |  | Multiple studies | Multiple | Mixed | 2006 |  |
| Glutathione S-transferase theta 1 (GSTT1), deletion | 22q11 | Multiple studies | Multiple | Mixed | 2009 |  |
|  |  | Multiple studies | Multiple | Mixed | 2006 |  |
|  |  |  |  |  |  |  |

| **Additional asthma candidate genes (not listed in tables above). identified in Weiss et al. Asthma genetics and genomics 2009. Current Opinion in Genetics & Development 2009, 19:279–282.**  **This review identified all asthma candidate genes published between 9/1/2005 and 7/1/2008 supplemented with genes identified through 2005 in a previous review (Ober C and Hoffjan S. Asthma genetics 2006: The long and winding road to gene discovery. *Genes and immunity* 2006;7:95-100).** | | | | |
| --- | --- | --- | --- | --- |
| ADAM metallopeptidase domain 33 (ADAM33) | *For details about references to genes in this table, please see Supplementary data to Weiss et al. Current Opinion in Genetics & Development 2009, 19:279–282.* | | | |
| Beta-2 adrenergic receptor (ADRB2) |  |  |  |  |
| Chemokine (C-C motif) ligand 11 (CCL11, eotaxin) |  |  |  |  |
| Chemokine (C-C motif) ligand 24 (CCL24, eotaxin-2) |  |  |  |  |
| Chemokine (C-C motif) ligand 5 (CCL5, Rantes) |  |  |  |  |
| CD14 molecule (CD14) |  |  |  |  |
| Chitinase 3-like 1 (CHI3L1) |  |  |  |  |
| Cytotoxic T-lymphocyte-associated protein 4 (CTLA4) |  |  |  |  |
| Chemokine (C-X3-C motif) receptor 1 (CX3CR1) |  |  |  |  |
| Cysteinyl leukotriene receptor 2 (CYSLTR2) |  |  |  |  |
| Endothelin 1 (EDN1) |  |  |  |  |
| G-protein coupled receptor 154 (GPR154, also neuropeptid receptor- NPSR)* |  |  |  |  |
| Glutathione-S transferase p1 (GSTP1) |  |  |  |  |
| Hepatitis A virus cellular recptor 1 (HAVCR1) |  |  |  |  |
| Interferon gamma (IFNG) |  |  |  |  |
| Interleukin 10 (IL10) |  |  |  |  |
| Interleukin 4 (IL4) |  |  |  |  |
| Interleukin 4 Receptor(IL4R) |  |  |  |  |
| Inosytol polyphosphate-4-phosphatase, type 1 (INPP4A) |  |  |  |  |
| Interleukin-1 receptor associated kinase 3 (IRAK-3, IRAK-M) |  |  |  |  |
| Integrin, β-3 (ITGB3) |  |  |  |  |
| Lymphotaxin alpha (LTA) |  |  |  |  |
| Membrane-spanning 4 domains, sub-family A, member 2 (MS4A2, also F_c_ fragment of IgE, high affinity I, receptor for, β polypeptide, FCER1β) |  |  |  |  |
| Myosin light chain kinase (MYLK) |  |  |  |  |
| N-acetyltransferase 2 (NAT2) |  |  |  |  |
| Nucleotide-binding oligomerization domain containing 1 (NOD1) |  |  |  |  |
| Nitric oxide synthase 3 (NOS3) |  |  |  |  |
| Natriuretic peptide precursor A (NPPA) |  |  |  |  |
| Phospholipase A2, group 7 (PLA2G7, also platelet activating factor acetylhydrolase, PAFAH) |  |  |  |  |
| PHD finger protein 11 (PHF11) |  |  |  |  |
| Prostaglandin D2 receptor (PTGDR) |  |  |  |  |
| Thromboxane A2 receptor (TBXA2R) |  |  |  |  |
| Toll-like receptor 4 (TLR4) |  |  |  |  |
| Toll-like receptor 9 (TLR9) |  |  |  |  |
| Transforming growth factor, β1 (TGFB1) |  |  |  |  |
| Tumor necrosis factor alpha (TNFα) |  |  |  |  |
| Uteroglobin (UGB, also Clara-cell specific 10kd protein, CC10, SCGB1A1) |  |  |  |  |
| * GPRA / NPSR1 is not represented on the Affy133 plus chip and therefore not included in the analyses. | | | | |

| **Table E2. Gene expression analysis of specific asthma genes and evidence for differential expression during human lung development** | | | | | |
| --- | --- | --- | --- | --- | --- |
| Gene symbol | Probe id | Average expression | p-value for differential expression | Adjusted p-value* | Beta coefficient† |
| *NOD1* | 221073_s_at | 7.62 | 3.99E-10 | 6.98E-08 | 0.012 |
| *EDN1* | 222802_at | 9.31 | 2.29E-08 | 1.57E-06 | 0.026 |
| *EDN1* | 218995_s_at | 8.63 | 8.61E-08 | 4.35E-06 | 0.019 |
| *ROBO1* | 213194_at | 10.17 | 4.04E-07 | 1.49E-05 | -0.009 |
| *IL4R* | 203233_at | 7.28 | 1.09E-06 | 3.35E-05 | 0.010 |
| *RORA* | 226682_at | 8.24 | 1.14E-06 | 3.49E-05 | 0.022 |
| *RORA* | 236266_at | 5.16 | 1.90E-06 | 5.23E-05 | 0.011 |
| *HPCAL1* | 212552_at | 9.36 | 1.99E-06 | 5.41E-05 | 0.012 |
| *HLA-DQB1* | 212998_x_at | 4.75 | 7.35E-06 | 1.55E-04 | 0.012 |
| *PLAUR* | 210845_s_at | 5.90 | 1.00E-05 | 1.99E-04 | -0.008 |
| *IL2RB* | 205291_at | 6.54 | 1.34E-05 | 2.50E-04 | 0.007 |
| *CCL5* | 204655_at | 4.69 | 1.43E-05 | 2.63E-04 | 0.008 |
| *HPCAL1* | 205462_s_at | 7.28 | 2.88E-05 | 4.58E-04 | 0.013 |
| *TLR10* | 223751_x_at | 4.09 | 3.15E-05 | 4.93E-04 | 0.005 |
| *PDE10A* | 205501_at | 6.42 | 4.30E-05 | 6.32E-04 | -0.011 |
| *CCL5* | 1405_i_at | 4.13 | 7.69E-05 | 9.92E-04 | 0.008 |
| *PLAUR* | 211924_s_at | 6.37 | 8.32E-05 | 1.05E-03 | -0.006 |
| *NOD1* | 224190_x_at | 6.50 | 1.01E-04 | 1.23E-03 | 0.008 |
| *IRAK3* | 213817_at | 7.46 | 1.10E-04 | 1.31E-03 | -0.011 |
| *HLA-DQB1* | 211656_x_at | 6.10 | 1.10E-04 | 1.31E-03 | 0.008 |
| *RORA* | 210426_x_at | 4.64 | 1.11E-04 | 1.31E-03 | 0.018 |
| *INPP4A* | 208363_s_at | 4.57 | 1.39E-04 | 1.57E-03 | -0.005 |
| *IL18* | 206295_at | 3.25 | 1.45E-04 | 1.62E-03 | 0.004 |
| *CCL11* | 210133_at | 4.66 | 1.52E-04 | 1.69E-03 | 0.006 |
| *STAT6* | 201331_s_at | 8.82 | 1.64E-04 | 1.80E-03 | 0.007 |
| *CHIA* | 220630_s_at | 4.89 | 1.67E-04 | 1.82E-03 | 0.009 |
| *TBXA2R* | 207555_s_at | 5.67 | 1.81E-04 | 1.94E-03 | -0.007 |
| *HLA-G* | 211529_x_at | 9.58 | 2.64E-04 | 2.61E-03 | 0.010 |
| *IL4R* | 242743_at | 5.19 | 3.82E-04 | 3.48E-03 | 0.008 |
| *OPN3* | 224392_s_at | 6.01 | 3.98E-04 | 3.59E-03 | -0.008 |
| *TBXA2R* | 336_at | 6.17 | 4.03E-04 | 3.63E-03 | 0.005 |
| *HLA-DQB1* | 211654_x_at | 5.89 | 5.16E-04 | 4.40E-03 | 0.009 |
| *NOS1* | 1560974_s_at | 4.49 | 5.49E-04 | 4.61E-03 | 0.005 |
| *HLA-G* | 211528_x_at | 9.65 | 6.23E-04 | 5.08E-03 | 0.008 |
| *PTGDR* | 234165_at | 3.51 | 6.95E-04 | 5.53E-03 | 0.003 |
| *CD86* | 205686_s_at | 3.64 | 6.97E-04 | 5.55E-03 | 0.003 |
| *CD86* | 210895_s_at | 4.04 | 7.10E-04 | 5.63E-03 | 0.005 |
| *WDR36* | 226180_at | 7.96 | 1.11E-03 | 7.86E-03 | -0.005 |
| *RORA* | 210479_s_at | 5.00 | 1.20E-03 | 8.37E-03 | 0.016 |
| *PRNP* | 1556190_s_at | 3.69 | 1.29E-03 | 8.87E-03 | -0.003 |
| *OPN3* | 219032_x_at | 8.40 | 1.33E-03 | 9.06E-03 | -0.005 |
| *PCDH1* | 203918_at | 6.47 | 1.52E-03 | 1.00E-02 | 0.006 |
| *DENND1B* | 1552835_at | 3.67 | 1.71E-03 | 1.10E-02 | 0.003 |
| *HLA-DQB1* | 209823_x_at | 5.24 | 2.53E-03 | 1.48E-02 | 0.007 |
| *SERPINE1* | 202627_s_at | 5.82 | 2.69E-03 | 1.56E-02 | 0.021 |
| *HLA-G* | 211530_x_at | 8.37 | 2.77E-03 | 1.59E-02 | 0.007 |
| *PDE10A* | 211170_s_at | 3.53 | 2.89E-03 | 1.64E-02 | -0.003 |
| *VDR* | 204254_s_at | 4.71 | 3.07E-03 | 1.72E-02 | 0.007 |
| *PDE4D* | 210837_s_at | 3.76 | 3.56E-03 | 1.92E-02 | 0.006 |
| *HLA-DQB1* | 209480_at | 3.86 | 3.83E-03 | 2.03E-02 | 0.009 |
| *RORA* | 1562682_at | 3.70 | 4.17E-03 | 2.16E-02 | 0.003 |
| *RORA* | 235567_at | 5.98 | 4.20E-03 | 2.18E-02 | 0.014 |
| *TNF* | 207113_s_at | 4.76 | 4.53E-03 | 2.30E-02 | 0.004 |
| *TLE4* | 233575_s_at | 4.28 | 4.57E-03 | 2.32E-02 | -0.004 |
| *PDE4D* | 204491_at | 7.55 | 4.76E-03 | 2.39E-02 | 0.005 |
| *DPP10* | 228598_at | 4.70 | 5.26E-03 | 2.58E-02 | -0.015 |
| *NOS1* | 207310_s_at | 4.51 | 5.35E-03 | 2.61E-02 | 0.004 |
| *HLA-DQB1* | 212999_x_at | 4.09 | 5.86E-03 | 2.79E-02 | 0.007 |
| *ARG2* | 203946_s_at | 3.91 | 6.11E-03 | 2.88E-02 | -0.006 |
| *NOS3* | 205581_s_at | 4.81 | 6.49E-03 | 3.02E-02 | -0.005 |
| *NOS1* | 239132_at | 4.26 | 6.63E-03 | 3.07E-02 | 0.005 |
| *CD86* | 205685_at | 4.29 | 8.47E-03 | 3.67E-02 | 0.003 |
| *IL13* | 207844_at | 4.39 | 8.85E-03 | 3.80E-02 | -0.003 |
| *SCGB1A1* | 205725_at | 6.29 | 8.95E-03 | 3.83E-02 | 0.017 |
| *TSLP* | 235737_at | 3.60 | 9.62E-03 | 4.04E-02 | 0.005 |
| *CTLA4* | 221331_x_at | 4.23 | 9.98E-03 | 4.16E-02 | 0.004 |
| *TBXA2R* | 207554_x_at | 5.50 | 1.01E-02 | 4.20E-02 | 0.004 |
| *IL12B* | 207901_at | 3.21 | 1.05E-02 | 4.31E-02 | 0.002 |
| *CYSLTR2* | 220813_at | 4.45 | 1.09E-02 | 4.46E-02 | 0.003 |
| *TLE4* | 216997_x_at | 4.38 | 1.10E-02 | 4.47E-02 | -0.006 |
| *PDE4D* | 210836_x_at | 4.09 | 1.12E-02 | 4.52E-02 | 0.005 |
| *MYLK* | 1568770_at | 4.54 | 1.28E-02 | 5.01E-02 | 0.004 |
| *IL1RL1* | 234066_at | 4.70 | 1.34E-02 | 5.18E-02 | 0.004 |
| *GSDMB* | 215659_at | 4.49 | 1.40E-02 | 5.37E-02 | 0.007 |
| *ADRB2* | 206170_at | 4.96 | 1.41E-02 | 5.39E-02 | 0.006 |
| *DENND1B* | 1564164_at | 6.14 | 1.45E-02 | 5.51E-02 | 0.008 |
| *MYLK* | 1563466_at | 4.54 | 1.68E-02 | 6.13E-02 | -0.004 |
| *PDE4D* | 211840_s_at | 4.48 | 1.78E-02 | 6.40E-02 | 0.004 |
| *GPR44* | 216464_x_at | 4.00 | 1.93E-02 | 6.82E-02 | -0.003 |
| *GSTP1* | 200824_at | 10.39 | 2.21E-02 | 7.55E-02 | -0.004 |
| *RAD50* | 209349_at | 5.90 | 2.34E-02 | 7.89E-02 | -0.005 |
| *MYLK* | 1569956_at | 4.49 | 2.38E-02 | 7.99E-02 | -0.004 |
| *PDE4D* | 1554717_a_at | 3.97 | 2.61E-02 | 8.52E-02 | 0.003 |
| *HLA-G* | 210514_x_at | 8.06 | 2.62E-02 | 8.56E-02 | 0.006 |
| *RORA* | 241760_x_at | 3.07 | 2.99E-02 | 9.44E-02 | 0.002 |
| *EDN1* | 1564630_at | 6.04 | 3.16E-02 | 9.84E-02 | 0.007 |
| *ORMDL3* | 235136_at | 4.83 | 3.31E-02 | 1.02E-01 | -0.007 |
| *VDR* | 213692_s_at | 4.29 | 3.47E-02 | 1.05E-01 | 0.003 |
| *NOS1* | 207309_at | 4.29 | 3.58E-02 | 1.08E-01 | 0.004 |
| *IRAK3* | 1568830_at | 4.70 | 4.56E-02 | 1.28E-01 | 0.005 |
| *HAVCR1* | 207052_at | 3.40 | 4.58E-02 | 1.29E-01 | 0.002 |
| *PTPRD* | 242493_at | 3.39 | 4.68E-02 | 1.31E-01 | 0.002 |
| *ADAM33* | 233868_x_at | 7.33 | 4.83E-02 | 1.34E-01 | 0.006 |
| *PTPRD* | 214043_at | 9.92 | 4.98E-02 | 1.37E-01 | 0.003 |
| *SPINK5* | 205185_at | 5.46 | 5.38E-02 | 1.44E-01 | 0.010 |
| *SERPINE1* | 202628_s_at | 5.51 | 5.82E-02 | 1.53E-01 | 0.007 |
| *STAT6* | 201332_s_at | 6.18 | 5.83E-02 | 1.53E-01 | 0.006 |
| *SMAD3* | 205396_at | 5.62 | 6.25E-02 | 1.61E-01 | -0.004 |
| *DARC* | 208335_s_at | 5.56 | 6.63E-02 | 1.68E-01 | 0.011 |
| *TBX21* | 220684_at | 5.03 | 6.81E-02 | 1.71E-01 | 0.003 |
| *SLC22A5* | 205074_at | 6.58 | 6.90E-02 | 1.72E-01 | 0.003 |
| *TLE4* | 235765_at | 5.75 | 6.93E-02 | 1.73E-01 | -0.004 |
| *ARG2* | 203945_at | 6.02 | 7.28E-02 | 1.79E-01 | -0.004 |
| *LTA* | 206975_at | 4.40 | 7.33E-02 | 1.80E-01 | 0.002 |
| *IL1RL1* | 207526_s_at | 3.94 | 7.55E-02 | 1.84E-01 | 0.002 |
| *CYP2R1* | 207786_at | 5.13 | 8.41E-02 | 1.98E-01 | -0.003 |
| *TLR10* | 223750_s_at | 3.79 | 1.02E-01 | 2.27E-01 | 0.001 |
| *VDR* | 204255_s_at | 3.85 | 1.05E-01 | 2.31E-01 | 0.002 |
| *CRB1* | 244403_at | 3.24 | 1.06E-01 | 2.33E-01 | 0.001 |
| *PTGDR* | 215894_at | 3.32 | 1.24E-01 | 2.60E-01 | 0.001 |
| *PRNP* | 201300_s_at | 9.41 | 1.24E-01 | 2.60E-01 | 0.004 |
| *INPP4A* | 204553_x_at | 5.80 | 1.25E-01 | 2.62E-01 | -0.002 |
| *INPP4A* | 204552_at | 5.74 | 1.29E-01 | 2.68E-01 | -0.004 |
| *TLE4* | 204872_at | 8.21 | 1.32E-01 | 2.72E-01 | 0.003 |
| *HLA-DQB1* | 210747_at | 3.41 | 1.36E-01 | 2.77E-01 | 0.001 |
| *CCL2* | 216598_s_at | 8.15 | 1.36E-01 | 2.78E-01 | 0.012 |
| *GBE1* | 203282_at | 7.50 | 1.46E-01 | 2.91E-01 | 0.006 |
| *RORA* | 240951_at | 3.40 | 1.52E-01 | 3.00E-01 | 0.001 |
| *TGFB1* | 203084_at | 4.31 | 1.56E-01 | 3.05E-01 | -0.002 |
| *TGFB1* | 203085_s_at | 6.26 | 1.63E-01 | 3.15E-01 | 0.005 |
| *TLR8* | 229560_at | 3.52 | 1.65E-01 | 3.18E-01 | 0.003 |
| *PEX19* | 201707_at | 7.91 | 1.77E-01 | 3.33E-01 | 0.002 |
| *CHI3L1* | 209395_at | 3.71 | 1.77E-01 | 3.33E-01 | 0.005 |
| *SMAD3* | 205398_s_at | 6.45 | 1.78E-01 | 3.35E-01 | -0.002 |
| *MYLK* | 224823_at | 11.97 | 1.80E-01 | 3.37E-01 | -0.001 |
| *CHI3L1* | 209396_s_at | 3.53 | 1.80E-01 | 3.38E-01 | 0.003 |
| *MYLK* | 202555_s_at | 11.83 | 1.83E-01 | 3.42E-01 | 0.002 |
| *NPPA* | 209957_s_at | 5.69 | 1.84E-01 | 3.43E-01 | 0.002 |
| *IL17A* | 208402_at | 4.07 | 1.86E-01 | 3.45E-01 | 0.001 |
| *INPP4A* | 227087_at | 8.36 | 1.93E-01 | 3.54E-01 | -0.002 |
| *DENND1B* | 219696_at | 8.07 | 1.96E-01 | 3.58E-01 | 0.003 |
| *SMAD3* | 205397_x_at | 5.61 | 2.03E-01 | 3.66E-01 | -0.002 |
| *IL1RL1* | 242809_at | 5.56 | 2.11E-01 | 3.76E-01 | 0.002 |
| *GSDMB* | 219233_s_at | 6.25 | 2.23E-01 | 3.91E-01 | 0.003 |
| *SERPINE1* | 1568765_at | 3.48 | 2.38E-01 | 4.08E-01 | 0.002 |
| *NOS2* | 210037_s_at | 4.70 | 2.48E-01 | 4.19E-01 | 0.001 |
| *CYSLTR1* | 231747_at | 4.47 | 2.60E-01 | 4.32E-01 | -0.002 |
| *NQO1* | 210519_s_at | 5.23 | 2.79E-01 | 4.54E-01 | -0.002 |
| *CCL5* | 1555759_a_at | 6.49 | 2.82E-01 | 4.57E-01 | 0.002 |
| *SMAD3* | 218284_at | 8.11 | 2.88E-01 | 4.63E-01 | 0.002 |
| *CCL26* | 223710_at | 3.82 | 2.93E-01 | 4.68E-01 | 0.001 |
| *CHI3L1* | 216546_s_at | 4.46 | 3.05E-01 | 4.81E-01 | 0.002 |
| *IL33* | 209821_at | 8.47 | 3.05E-01 | 4.81E-01 | 0.002 |
| *ADRA1B* | 207589_at | 4.10 | 3.16E-01 | 4.91E-01 | -0.001 |
| *IL10* | 207433_at | 3.67 | 3.40E-01 | 5.17E-01 | 0.001 |
| *ADAM33* | 232570_s_at | 5.47 | 3.41E-01 | 5.18E-01 | 0.002 |
| *ITGB3* | 204625_s_at | 4.15 | 3.57E-01 | 5.34E-01 | -0.001 |
| *DENND1B* | 1557309_at | 3.60 | 3.73E-01 | 5.49E-01 | -0.001 |
| *PLA2G7* | 206214_at | 3.34 | 3.89E-01 | 5.64E-01 | 0.001 |
| *NQO1* | 201468_s_at | 6.35 | 3.89E-01 | 5.64E-01 | -0.002 |
| *ORMDL3* | 223259_at | 7.39 | 3.89E-01 | 5.64E-01 | 0.001 |
| *CRB1* | 220522_at | 4.40 | 3.99E-01 | 5.73E-01 | 0.001 |
| *CYP24A1* | 206504_at | 3.12 | 4.16E-01 | 5.88E-01 | 0.001 |
| *CTLA4* | 234362_s_at | 4.02 | 4.18E-01 | 5.90E-01 | 0.002 |
| *INPP4A* | 235695_at | 4.99 | 4.34E-01 | 6.04E-01 | 0.002 |
| *CX3CR1* | 205898_at | 6.17 | 4.44E-01 | 6.14E-01 | 0.004 |
| *MYB* | 204798_at | 5.73 | 4.49E-01 | 6.18E-01 | 0.001 |
| *PHF11* | 221816_s_at | 8.69 | 4.49E-01 | 6.18E-01 | 0.001 |
| *CTLA4* | 236341_at | 3.54 | 4.57E-01 | 6.25E-01 | 0.001 |
| *CTLA4* | 231794_at | 3.77 | 4.59E-01 | 6.27E-01 | -0.001 |
| *PDE10A* | 211171_s_at | 4.14 | 4.78E-01 | 6.43E-01 | 0.001 |
| *ITGB3* | 211579_at | 3.54 | 4.82E-01 | 6.47E-01 | -0.001 |
| *FLG* | 215704_at | 3.21 | 4.83E-01 | 6.47E-01 | 0.000 |
| *IL4* | 207539_s_at | 3.39 | 4.84E-01 | 6.48E-01 | 0.001 |
| *RORA* | 240163_at | 3.59 | 4.91E-01 | 6.54E-01 | 0.001 |
| *TLE4* | 214688_at | 3.93 | 5.00E-01 | 6.61E-01 | 0.001 |
| *NQO1* | 201467_s_at | 4.42 | 5.08E-01 | 6.68E-01 | -0.001 |
| *RORA* | 239550_at | 4.09 | 5.22E-01 | 6.80E-01 | 0.001 |
| *IRAK3* | 220034_at | 5.83 | 5.33E-01 | 6.89E-01 | 0.002 |
| *VDR* | 204253_s_at | 5.45 | 5.42E-01 | 6.96E-01 | -0.001 |
| *ITGB3* | 215240_at | 3.38 | 5.51E-01 | 7.03E-01 | 0.000 |
| *PTPRD* | 213362_at | 9.68 | 5.59E-01 | 7.09E-01 | 0.001 |
| *CYSLTR1* | 230866_at | 3.74 | 5.71E-01 | 7.19E-01 | -0.001 |
| *CX3CR1* | 1568934_at | 4.17 | 5.72E-01 | 7.19E-01 | -0.001 |
| *PEX19* | 201706_s_at | 8.39 | 5.87E-01 | 7.32E-01 | -0.001 |
| *TLR4* | 1552798_a_at | 5.13 | 5.90E-01 | 7.34E-01 | 0.001 |
| *ITGB3* | 204626_s_at | 5.42 | 5.91E-01 | 7.35E-01 | 0.001 |
| *TLR4* | 224341_x_at | 4.36 | 5.94E-01 | 7.37E-01 | -0.001 |
| *PCDH1* | 215277_at | 4.35 | 5.96E-01 | 7.38E-01 | 0.001 |
| *NOS1* | 240911_at | 4.50 | 5.99E-01 | 7.41E-01 | 0.001 |
| *TBXA2R* | 211590_x_at | 5.69 | 6.03E-01 | 7.43E-01 | 0.001 |
| *GSTT1* | 203815_at | 6.77 | 6.04E-01 | 7.44E-01 | 0.006 |
| *SLC22A5* | 239615_at | 3.81 | 6.12E-01 | 7.50E-01 | -0.001 |
| *IL4* | 207538_at | 3.58 | 6.18E-01 | 7.55E-01 | 0.001 |
| *TLR8* | 220832_at | 3.70 | 6.21E-01 | 7.57E-01 | 0.001 |
| *GSTM1* | 215333_x_at | 8.50 | 6.40E-01 | 7.71E-01 | -0.002 |
| *PTGDR* | 215937_at | 3.55 | 6.62E-01 | 7.88E-01 | -0.001 |
| *PTPRD* | 205712_at | 7.97 | 6.75E-01 | 7.96E-01 | -0.001 |
| *CCL24* | 221463_at | 4.47 | 6.79E-01 | 8.00E-01 | -0.001 |
| *TLR9* | 223903_at | 4.51 | 7.12E-01 | 8.23E-01 | -0.001 |
| *BDNF* | 206382_s_at | 3.72 | 7.27E-01 | 8.33E-01 | 0.000 |
| *MS4A2* | 207497_s_at | 3.58 | 7.31E-01 | 8.37E-01 | 0.000 |
| *IL18R1* | 206618_at | 4.59 | 7.51E-01 | 8.50E-01 | 0.001 |
| *PLAUR* | 214866_at | 4.83 | 7.64E-01 | 8.58E-01 | 0.000 |
| *CTLA4* | 234895_at | 5.29 | 7.67E-01 | 8.61E-01 | 0.000 |
| *PHF11* | 242060_x_at | 4.35 | 7.67E-01 | 8.61E-01 | 0.001 |
| *WDR36* | 238677_at | 4.73 | 7.96E-01 | 8.80E-01 | -0.001 |
| *IL1RL1* | 210442_at | 4.93 | 8.13E-01 | 8.90E-01 | 0.000 |
| *TLR4* | 232068_s_at | 3.44 | 8.42E-01 | 9.09E-01 | 0.000 |
| *ITGB3* | 204628_s_at | 5.33 | 8.46E-01 | 9.11E-01 | 0.000 |
| *GPR44* | 206361_at | 5.28 | 8.47E-01 | 9.12E-01 | 0.000 |
| *ITGB3* | 216261_at | 4.14 | 8.48E-01 | 9.12E-01 | 0.000 |
| *GSTT1* | 232193_at | 4.93 | 8.67E-01 | 9.24E-01 | 0.001 |
| *TLR4* | 221060_s_at | 5.10 | 8.71E-01 | 9.26E-01 | 0.000 |
| *MS4A2* | 207496_at | 3.29 | 8.77E-01 | 9.30E-01 | 0.000 |
| *MYB* | 215152_at | 3.20 | 8.87E-01 | 9.35E-01 | 0.000 |
| *NAT2* | 206797_at | 4.95 | 8.89E-01 | 9.37E-01 | 0.000 |
| *PDE4D* | 228962_at | 5.29 | 8.91E-01 | 9.38E-01 | 0.000 |
| *IFNG* | 210354_at | 3.82 | 8.93E-01 | 9.39E-01 | 0.000 |
| *INPP4A* | 208364_at | 4.33 | 8.94E-01 | 9.40E-01 | 0.000 |
| *CMA1* | 214533_at | 3.86 | 9.03E-01 | 9.45E-01 | 0.000 |
| *IL17A* | 216876_s_at | 3.26 | 9.07E-01 | 9.47E-01 | 0.000 |
| *CYP2R1* | 227109_at | 7.19 | 9.48E-01 | 9.71E-01 | 0.000 |
| *PRNP* | 215707_s_at | 7.13 | 9.65E-01 | 9.81E-01 | 0.000 |
| *RAD50* | 208393_s_at | 7.19 | 9.69E-01 | 9.83E-01 | 0.000 |
| *GSTM1* | 204550_x_at | 8.13 | 9.70E-01 | 9.83E-01 | 0.000 |
| *ITGB3* | 204627_s_at | 4.36 | 9.72E-01 | 9.84E-01 | 0.000 |
| *CD14* | 201743_at | 6.93 | 9.85E-01 | 9.92E-01 | 0.000 |
| *BDNF* | 239367_at | 4.46 | 9.89E-01 | 9.94E-01 | 0.000 |
| *CYSLTR1* | 216288_at | 3.28 | 9.93E-01 | 9.96E-01 | 0.000 |
| *Adjusted according to the BH model. † The beta coefficient corresponds to the mean change in gene expression per day during the studied period (7-26 weeks of gestational age). | | | | | |

| **Table E3. Gene expression analysis of specific asthma genes and evidence for differential expression during human lung development; pseudoglandular vs canalicular period (top hits presented)** | | | |
| --- | --- | --- | --- |
| **Human gene symbol** | **Probe id** | **Average expression** | **Adjusted p-value for differential expression*** |
| *ROBO1* | 213194_at | 10,2 | 3,0E-04 |
| *EDN1* | 222802_at | 9,3 | 7,1E-04 |
| *NOD1* | 221073_s_at | 7,6 | 7,8E-04 |
| *IL4R* | 203233_at | 7,3 | 1,9E-03 |
| *CHIA* | 220630_s_at | 4,9 | 2,2E-03 |
| *PDE10A* | 205501_at | 6,4 | 2,3E-03 |
| *IRAK3* | 213817_at | 7,5 | 3,1E-03 |
| *EDN1* | 218995_s_at | 8,6 | 3,6E-03 |
| *RORA* | 236266_at | 5,2 | 3,8E-03 |
| *IL18* | 206295_at | 3,2 | 4,4E-03 |
| *DENND1B* | 1552835_at | 3,7 | 6,9E-03 |
| *TBXA2R* | 207555_s_at | 5,7 | 7,1E-03 |
| *INPP4A* | 208363_s_at | 4,6 | 7,7E-03 |
| *NOS1* | 1560974_s_at | 4,5 | 7,9E-03 |
| *TLR10* | 223751_x_at | 4,1 | 9,2E-03 |
| *IL4R* | 242743_at | 5,2 | 1,0E-02 |
| ***Test for difference between the two stages, adjusted according to BH. | | | |

**Figure E1. Expression of EDN1 222802_at in human lungs during the pseudoglandular and canalicular stages (p=7.1E-04 for differential expression).**

| **Table E4. Significant bioontologic attributes to genes with evidence of differential expression during fetal and murine lung development*** | | | |
| --- | --- | --- | --- |
| **Category** | **Term** | **Genes** | **Adjusted p-value** |
| GOTERM_BP_FAT | GO:0001817~regulation of cytokine production | *IRAK3, CD86, NOD1, TNF, IL18, SCGB1A1* | 1,18E-03 |
| GOTERM_BP_FAT | GO:0050865~regulation of cell activation | *STAT6, CD86, IL18, IL4R, RORA, SCGB1A1* | 1,46E-03 |
| SP_PIR_KEYWORDS | disulfide bond | *CD86, CHIA, TNF, ROBO1, IL4R, EDN1, PRNP, CCL5, SCGB1A1, HLA-G, PLAUR* | 1,91E-03 |
| SP_PIR_KEYWORDS | Secreted | *CHIA, TNF, IL18, IL4R, EDN1, SERPINE1, CCL5, SCGB1A1, PLAUR* | 2,10E-03 |
| UP_SEQ_FEATURE | signal peptide | *PCDH1, CD86, CHIA, ROBO1, IL4R, EDN1, SERPINE1, PRNP, CCL5, SCGB1A1, HLA-G, PLAUR* | 2,17E-03 |
| GOTERM_BP_FAT | GO:0002694~regulation of leukocyte activation | *STAT6, CD86, IL18, IL4R, RORA, SCGB1A1* | 2,24E-03 |
| SP_PIR_KEYWORDS | Signal | *PCDH1, CD86, CHIA, ROBO1, IL4R, EDN1, SERPINE1, PRNP, CCL5, SCGB1A1, HLA-G, PLAUR* | 3,14E-03 |
| UP_SEQ_FEATURE | disulfide bond | *CD86, CHIA, TNF, ROBO1, IL4R, EDN1, PRNP, CCL5, SCGB1A1, HLA-G, PLAUR* | 3,60E-03 |
| OMIM_DISEASE | Asthma, susceptibility to | *TNF, SCGB1A1, HLA-G* | 7,37E-03 |
| GOTERM_BP_FAT | GO:0051249~regulation of lymphocyte activation | *STAT6, CD86, IL18, IL4R, SCGB1A1* | 1,02E-02 |
| GOTERM_BP_FAT | GO:0002828~regulation of T-helper 2 type immune response | *STAT6, CD86, IL4R* | 1,23E-02 |
| GOTERM_BP_FAT | GO:0051241~negative regulation of multicellular organismal process | *IRAK3, TNF, EDN1, SERPINE1, SCGB1A1* | 1,25E-02 |
| GOTERM_CC_FAT | GO:0005576~extracellular region | *CHIA, TNF, IL18, IL4R, EDN1, SERPINE1, CCL5, SCGB1A1, PLAUR* | 1,83E-02 |
| GOTERM_BP_FAT | GO:0006955~immune response | *CD86, CHIA, TNF, IL18, IL4R, CCL5, HLA-G* | 2,07E-02 |
| GOTERM_BP_FAT | GO:0002683~negative regulation of immune system process | *STAT6, IRAK3, IL4R, SCGB1A1* | 2,15E-02 |
| GOTERM_BP_FAT | GO:0051251~positive regulation of lymphocyte activation | *STAT6, CD86, IL18, IL4R* | 2,56E-02 |
| GOTERM_BP_FAT | GO:0051240~positive regulation of multicellular organismal process | *NOD1, TNF, IL18, EDN1, CCL5* | 2,69E-02 |
| GOTERM_BP_FAT | GO:0002684~positive regulation of immune system process | *STAT6, IRAK3, CD86, IL18, IL4R* | 2,71E-02 |
| KEGG_PATHWAY | hsa04621:NOD-like receptor signaling pathway | *NOD1, TNF, IL18, CCL5* | 2,72E-02 |
| GOTERM_BP_FAT | GO:0002696~positive regulation of leukocyte activation | *STAT6, CD86, IL18, IL4R* | 3,04E-02 |
| GOTERM_BP_FAT | GO:0050863~regulation of T cell activation | *CD86, IL18, IL4R, SCGB1A1* | 3,04E-02 |
| GOTERM_BP_FAT | GO:0051094~positive regulation of developmental process | *CD86, TNF, ROBO1, IL4R, CCL5* | 3,08E-02 |
| GOTERM_BP_FAT | GO:0050867~positive regulation of cell activation | *STAT6, CD86, IL18, IL4R* | 3,21E-02 |
| GOTERM_BP_FAT | GO:0042127~regulation of cell proliferation | *STAT6, CD86, TNF, IL18, EDN1, SERPINE1, SCGB1A1* | 3,29E-02 |
| * All 19 genes in Table 4 were included in ontology enrichment analyses using DAVID, and attributes with adjusted p-value <0.05 are here presented. | | | |

| **Table E5. Genes with evidence of differential expression during fetal and murine lung development* and their involvement in asthma pathogenesis supported by gene expression / protein in *human* and *murine* experimental studies** | | | | | |
| --- | --- | --- | --- | --- | --- |
| **Human gene symbol** | **Mouse gene**  **symbol** | **Involvement in asthma pathogenesis supported by gene expression / protein in *human* experimental studies** | **Reference, human data** | **Involvement in asthma pathogenesis supported by gene expression / protein in *murine* experimental studies** | **Reference, murine data** |
| *NOD1* | *Nod1* | *Yes* |  | *Yes* |  |
| *EDN1* | *Edn1* | *Yes* |  | *Yes* |  |
| *ROBO1* | *Robo1* | *No* | - | *No* | - |
| *IL4R* | *Il4ra* | *Yes* |  | *Yes* |  |
| *RORA* | *Rora* | *Yes* |  | *Yes* |  |
| *PLAUR* | *Plaur* | *Yes* |  | *No* | - |
| *CCL5* | *Ccl5* | *Yes* |  | *Yes* |  |
| *IRAK3* | *Irak3* | *No* | - | *No* | - |
| *IL18* | *Il18* | *Yes* |  | *Yes* |  |
| *STAT6* | *Stat6* | *Yes* |  | *Yes* |  |
| *CHIA* | *Chia* | *No* | - | *Yes* |  |
| *HLA-G* | *H2-M3* | *Yes* |  | *No* | - |
| *CD86* | *Cd86* | *Yes* |  | *Yes* |  |
| *PRNP* | *Prnp* | *Yes* |  | *No* | - |
| *PCDH1* | *Pcdh1* | *Yes* |  | *No* | - |
| *SERPINE1* | *Serpine1* | *Yes* |  | *Yes* |  |
| *TNF* | *Tnf* | *Yes* |  | *Yes* |  |
| *TLE4* | *Tle4* | *No* | - | *No* | - |
| *SCGB1A1* | *Scgb1a1* | *Yes* |  | *Yes* |  |
| * All 19 genes in Table 4 were included in ontology enrichment analyses using DAVID, and attributes with adjusted p-value <0.05 are here presented. | | | | | |

| **Table E6. Genes with at least one significant probe per gene in the human data and the A/J data set  using prenatal time points only*** | | | | |
| --- | --- | --- | --- | --- |
| Human gene symbol | Mouse gene symbol | Adjusted p-value, A/J data† | Adjusted p-value, human data† |  |
| *EDN1* | *Edn1* | 9,6E-03 | 4,4E-06 |  |
| *HPCAL1* | *Hpcal1* | 2,1E-05 | 5,4E-05 |  |
| *PLAUR* | *Plaur* | 7,5E-03 | 2,0E-04 |  |
| *HPCAL1* | *Hpcal1* | 2,1E-05 | 4,6E-04 |  |
| *PLAUR* | *Plaur* | 7,5E-03 | 1,1E-03 |  |
| *STAT6* | *Stat6* | 8,3E-04 | 1,8E-03 |  |
| *CHIA* | *Chia* | 8,7E-04 | 1,8E-03 |  |
| *IL4R* | *Il4ra* | 1,1E-04 | 3,5E-03 |  |
| *PRNP* | *Prnp* | 1,2E-03 | 8,9E-03 |  |
| *SERPINE1* | *Serpine1* | 1,1E-02 | 1,6E-02 |  |
| *SCGB1A1* | *Scgb1a1* | 9,1E-06 | 3,8E-02 |  |
| *N = 8 A/J samples from day 14 and day 17  †Adjusted p-value (B-H method) for differential expression over time. | | | |  |

**References**

1. Mathias RA, Grant AV, Rafaels N, Hand T, Gao L, Vergara C, Tsai YJ, Yang M, Campbell M, Foster C, Gao P, Togias A, Hansel NN, Diette G, Adkinson NF, Liu MC, Faruque M, Dunston GM, Watson HR, Bracken MB, Hoh J, Maul P, Maul T, Jedlicka AE, Murray T, Hetmanski JB, Ashworth R, Ongaco CM, Hetrick KN, Doheny KF *et al*: **A genome-wide association study on African-ancestry populations for asthma**. *J Allergy Clin Immunol* 2009, **November 10**.

2. Sleiman PM, Flory J, Imielinski M, Bradfield JP, Annaiah K, Willis-Owen SA, Wang K, Rafaels NM, Michel S, Bonnelykke K, Zhang H, Kim CE, Frackelton EC, Glessner JT, Hou C, Otieno FG, Santa E, Thomas K, Smith RM, Glaberson WR, Garris M, Chiavacci RM, Beaty TH, Ruczinski I, Orange JM, Allen J, Spergel JM, Grundmeier R, Mathias RA, Christie JD *et al*: **Variants of DENND1B associated with asthma in children**. *N Engl J Med* 2010, **362**(1):36-44.

3. Moffatt MF, Gut IG, Demenais F, Strachan DP, Bouzigon E, Heath S, von Mutius E, Farrall M, Lathrop M, Cookson WO, consortium. G: **A large-scale, consortium-based genomewide association study of asthma**. *N Engl J Med* 2010, **363**(13):1211-1221.

4. Moffatt MF, Kabesch M, Liang L, Dixon AL, Strachan D, Heath S, Depner M, von Berg A, Bufe A, Rietschel E, Heinzmann A, Simma B, Frischer T, Willis-Owen SA, Wong KC, Illig T, Vogelberg C, Weiland SK, von Mutius E, Abecasis GR, Farrall M, Gut IG, Lathrop GM, Cookson WO: **Genetic variants regulating ORMDL3 expression contribute to the risk of childhood asthma**. *Nature* 2007, **448**(7152):470-473.

5. Gudbjartsson DF, Bjornsdottir US, Halapi E, Helgadottir A, Sulem P, Jonsdottir GM, Thorleifsson G, Helgadottir H, Steinthorsdottir V, Stefansson H, Williams C, Hui J, Beilby J, Warrington NM, James A, Palmer LJ, Koppelman GH, Heinzmann A, Krueger M, Boezen HM, Wheatley A, Altmuller J, Shin HD, Uh ST, Cheong HS, Jonsdottir B, Gislason D, Park CS, Rasmussen LM, Porsbjerg C *et al*: **Sequence variants affecting eosinophil numbers associate with asthma and myocardial infarction**. *Nat Genet* 2009, **41**(3):342-347.

6. Himes BE, Hunninghake GM, Baurley JW, Rafaels NM, Sleiman P, Strachan DP, Wilk JB, Willis-Owen SA, Klanderman B, Lasky-Su J, Lazarus R, Murphy AJ, Soto-Quiros ME, Avila L, Beaty T, Mathias RA, Ruczinski I, Barnes KC, Celedon JC, Cookson WO, Gauderman WJ, Gilliland FD, Hakonarson H, Lange C, Moffatt MF, O'Connor GT, Raby BA, Silverman EK, Weiss ST: **Genome-wide association analysis identifies PDE4D as an asthma-susceptibility gene**. *Am J Hum Genet* 2009, **84**(5):581-593.

7. Li X, Howard TD, Zheng SL, Haselkorn T, Peters SP, Meyers DA, Bleecker ER: **Genome-wide association study of asthma identifies RAD50-IL13 and HLA-DR/DQ regions**. *J Allergy Clin Immunol* 2010, **125**(2):328-335 e311.

8. Hancock DB, Romieu I, Shi M, Sienra-Monge JJ, Wu H, Chiu GY, Li H, del Rio-Navarro BE, Willis-Owens SA, Weiss ST, Raby BA, Gao H, Eng C, Chapela R, Burchard EG, Tang H, Sullivan PF, London SJ: **Genome-wide association study implicates chromosome 9q21.31 as a susceptibility locus for asthma in mexican children**. *PLoS Genet* 2009, **5**(8):e1000623.

9. Salam MT, Islam T, Gauderman WJ, Gilliland FD: **Roles of arginase variants, atopy, and ozone in childhood asthma**. *J Allergy Clin Immunol* 2009, **123**(3):596-602, 602 e591-598.

10. Li H, Romieu I, Sienra-Monge JJ, Ramirez-Aguilar M, Estela Del Rio-Navarro B, Kistner EO, Gjessing HK, Lara-Sanchez Idel C, Chiu GY, London SJ: **Genetic polymorphisms in arginase I and II and childhood asthma and atopy**. *J Allergy Clin Immunol* 2006, **117**(1):119-126.

11. Zeilinger S, Pinto LA, Nockher WA, Depner M, Klopp N, Illig T, von Mutius E, Renz H, Kabesch M: **The effect of BDNF gene variants on asthma in German children**. *Allergy* 2009, **64**(12):1790-1794.

12. Szczepankiewicz A, Rose-Zerilli MJ, Barton SJ, Holgate ST, Holloway JW: **Association analysis of brain-derived neurotrophic factor gene polymorphisms in asthmatic families**. *Int Arch Allergy Immunol* 2009, **149**(4):343-349.

13. Szczepankiewicz A, Breborowicz A, Skibinska M, Wilkosc M, Tomaszewska M, Hauser J: **Association analysis of brain-derived neurotrophic factor gene polymorphisms in asthmatic children**. *Pediatr Allergy Immunol* 2007, **18**(4):293-297.

14. Bosse Y, Lemire M, Poon AH, Daley D, He JQ, Sandford A, White JH, James AL, Musk AW, Palmer LJ, Raby BA, Weiss ST, Kozyrskyj AL, Becker A, Hudson TJ, Laprise C: **Asthma and genes encoding components of the vitamin D pathway**. *Respir Res* 2009, **10**:98.

15. Lee JH, Moore JH, Park SW, Jang AS, Uh ST, Kim YH, Park CS, Park BL, Shin HD: **Genetic interactions model among Eotaxin gene polymorphisms in asthma**. *J Hum Genet* 2008, **53**(10):867-875.

16. Ober C, Hoffjan S: **Asthma genetics 2006: the long and winding road to gene discovery**. *Genes Immun* 2006, **7**(2):95-100.

17. Chelbi H, Ghadiri A, Lacheb J, Ghandil P, Hamzaoui K, Hamzaoui A, Combadiere C: **A polymorphism in the CCL2 chemokine gene is associated with asthma risk: a case-control and a family study in Tunisia**. *Genes Immun* 2008, **9**(7):575-581.

18. Szalai C, Kozma GT, Nagy A, Bojszko A, Krikovszky D, Szabo T, Falus A: **Polymorphism in the gene regulatory region of MCP-1 is associated with asthma susceptibility and severity**. *J Allergy Clin Immunol* 2001, **108**(3):375-381.

19. Seibold MA, Reese TA, Choudhry S, Salam MT, Beckman K, Eng C, Atakilit A, Meade K, Lenoir M, Watson HG, Thyne S, Kumar R, Weiss KB, Grammer LC, Avila P, Schleimer RP, Fahy JV, Rodriguez-Santana J, Rodriguez-Cintron W, Boot RG, Sheppard D, Gilliland FD, Locksley RM, Burchard EG: **Differential enzymatic activity of common haplotypic versions of the human acidic Mammalian chitinase protein**. *J Biol Chem* 2009, **284**(29):19650-19658.

20. Chatterjee R, Batra J, Das S, Sharma SK, Ghosh B: **Genetic association of acidic mammalian chitinase with atopic asthma and serum total IgE levels**. *J Allergy Clin Immunol* 2008, **122**(1):202-208, 208 e201-207.

21. Bierbaum S, Nickel R, Koch A, Lau S, Deichmann KA, Wahn U, Superti-Furga A, Heinzmann A: **Polymorphisms and haplotypes of acid mammalian chitinase are associated with bronchial asthma**. *Am J Respir Crit Care Med* 2005, **172**(12):1505-1509.

22. Hong X, Zhou H, Tsai HJ, Wang X, Liu X, Wang B, Xu X, Xu X: **Cysteinyl leukotriene receptor 1 gene variation and risk of asthma**. *Eur Respir J* 2009, **33**(1):42-48.

23. Sanz C, Isidro-Garcia M, Davila I, Moreno E, Laffond E, Lorente F: **Analysis of 927T> C CYSLTRI and -444A > C LTC4S polymorphisms in patients with asthma**. *J Investig Allergol Clin Immunol* 2006, **16**(6):331-337.

24. Vergara C, Tsai YJ, Grant AV, Rafaels N, Gao L, Hand T, Stockton M, Campbell M, Mercado D, Faruque M, Dunston G, Beaty TH, Oliveira RR, Ponte EV, Cruz AA, Carvalho E, Araujo MI, Watson H, Schleimer RP, Caraballo L, Nickel RG, Mathias RA, Barnes KC: **Gene encoding Duffy antigen/receptor for chemokines is associated with asthma and IgE in three populations**. *Am J Respir Crit Care Med* 2008, **178**(10):1017-1022.

25. van den Oord RA, Sheikh A: **Filaggrin gene defects and risk of developing allergic sensitisation and allergic disorders: systematic review and meta-analysis**. *Bmj* 2009, **339**:b2433.

26. Cameron L, Depner M, Kormann M, Klopp N, Illig T, von Mutius E, Kabesch M: **Genetic variation in CRTh2 influences development of allergic phenotypes**. *Allergy* 2009, **64**(10):1478-1485.

27. Wang J, Xu Y, Zhao H, Sui H, Liang H, Jiang X: **Genetic variations in chemoattractant receptor expressed on Th2 cells (CRTH2) is associated with asthma susceptibility in Chinese children**. *Mol Biol Rep* 2009, **36**(6):1549-1553.

28. Imada Y, Fujimoto M, Hirata K, Hirota T, Suzuki Y, Saito H, Matsumoto K, Akazawa A, Katsunuma T, Yoshihara S, Ebisawa M, Shibasaki M, Arinami T, Tamari M, Noguchi E: **Large scale genotyping study for asthma in the Japanese population**. *BMC Res Notes* 2009, **2**:54.

29. Wang JY, Shyur SD, Wang WH, Liou YH, Lin CG, Wu YJ, Wu LS: **The polymorphisms of interleukin 17A (IL17A) gene and its association with pediatric asthma in Taiwanese population**. *Allergy* 2009, **64**(7):1056-1060.

30. Zhou H, Hong X, Jiang S, Dong H, Xu X: **Analyses of associations between three positionally cloned asthma candidate genes and asthma or asthma-related phenotypes in a Chinese population**. *BMC Med Genet* 2009, **10**:123.

31. Nicolae D, Cox NJ, Lester LA, Schneider D, Tan Z, Billstrand C, Kuldanek S, Donfack J, Kogut P, Patel NM, Goodenbour J, Howard T, Wolf R, Koppelman GH, White SR, Parry R, Postma DS, Meyers D, Bleecker ER, Hunt JS, Solway J, Ober C: **Fine mapping and positional candidate studies identify HLA-G as an asthma susceptibility gene on chromosome 6p21**. *Am J Hum Genet* 2005, **76**(2):349-357.

32. Hattori T, Konno S, Hizawa N, Isada A, Takahashi A, Shimizu K, Gao P, Beaty TH, Barnes KC, Huang SK, Nishimura M: **Genetic variants in the mannose receptor gene (MRC1) are associated with asthma in two independent populations**. *Immunogenetics* 2009, **61**(11-12):731-738.

33. Islam T, Breton C, Salam MT, McConnell R, Wenten M, Gauderman WJ, Conti D, Van Den Berg D, Peters JM, Gilliland FD: **Role of inducible nitric oxide synthase in asthma risk and lung function growth during adolescence**. *Thorax* 2009, **65**(2):139-145.

34. Batra J, Pratap Singh T, Mabalirajan U, Sinha A, Prasad R, Ghosh B: **Association of inducible nitric oxide synthase with asthma severity, total serum immunoglobulin E and blood eosinophil levels**. *Thorax* 2007, **62**(1):16-22.

35. White JH, Chiano M, Wigglesworth M, Geske R, Riley J, White N, Hall S, Zhu G, Maurio F, Savage T, Anderson W, Cordy J, Ducceschi M, Vestbo J, Pillai SG: **Identification of a novel asthma susceptibility gene on chromosome 1qter and its functional evaluation**. *Hum Mol Genet* 2008, **17**(13):1890-1903.

36. Koppelman GH, Meyers DA, Howard TD, Zheng SL, Hawkins GA, Ampleford EJ, Xu J, Koning H, Bruinenberg M, Nolte IM, van Diemen CC, Boezen HM, Timens W, Whittaker PA, Stine OC, Barton SJ, Holloway JW, Holgate ST, Graves PE, Martinez FD, van Oosterhout AJ, Bleecker ER, Postma DS: **Identification of PCDH1 as a novel susceptibility gene for bronchial hyperresponsiveness**. *Am J Respir Crit Care Med* 2009, **180**(10):929-935.

37. Li YF, Tseng PJ, Lin CC, Hung CL, Lin SC, Su WC, Huang YL, Sung FC, Tai CK: **NAD(P)H: Quinone oxidoreductase 1, glutathione S-transferase M1, environmental tobacco smoke exposure, and childhood asthma**. *Mutat Res* 2009, **678**(1):53-58.

38. Castro-Giner F, Kunzli N, Jacquemin B, Forsberg B, de Cid R, Sunyer J, Jarvis D, Briggs D, Vienneau D, Norback D, Gonzalez JR, Guerra S, Janson C, Anto JM, Wjst M, Heinrich J, Estivill X, Kogevinas M: **Traffic-related air pollution, oxidative stress genes, and asthma (ECHRS)**. *Environ Health Perspect* 2009, **117**(12):1919-1924.

39. Millstein J CD, Gilliland FD, Gauderman WJ.: **A testing framework for identifying suseptibility genes in the presence of epistasis**. *Am J Hum Genet* 2006, **78**:15-27.

40. Barton SJ, Koppelman GH, Vonk JM, Browning CA, Nolte IM, Stewart CE, Bainbridge S, Mutch S, Rose-Zerilli MJ, Postma DS, Maniatis N, Henry AP, Hall IP, Holgate ST, Tighe P, Holloway JW, Sayers I: **PLAUR polymorphisms are associated with asthma, PLAUR levels, and lung function decline**. *J Allergy Clin Immunol* 2009, **123**(6):1391-1400 e1317.

41. Liu Q, Xia Y, Zhang W, Li J, Wang P, Li H, Wei C, Gong Y: **A functional polymorphism in the SPINK5 gene is associated with asthma in a Chinese Han Population**. *BMC Med Genet* 2009, **10**:59.

42. Kowal K, Bodzenta-Lukaszyk A, Pampuch A, Szmitkowski M, Zukowski S, Donati MB, Iacoviello L: **Analysis of -675 4 g/5 G SERPINE1 and C-159T CD14 polymorphisms in house dust mite-allergic asthma patients**. *J Investig Allergol Clin Immunol* 2008, **18**(4):284-292.

43. Pampuch A, Kowal K, Bodzenta-Lukaszyk A, Di Castelnuovo A, Chyczewski L, Donati MB, Iacoviello L: **The -675 4G/5G plasminogen activator inhibitor-1 promoter polymorphism in house dust mite-sensitive allergic asthma patients**. *Allergy* 2006, **61**(2):234-238.

44. Buckova D, Izakovicova Holla L, Vacha J: **Polymorphism 4G/5G in the plasminogen activator inhibitor-1 (PAI-1) gene is associated with IgE-mediated allergic diseases and asthma in the Czech population**. *Allergy* 2002, **57**(5):446-448.

45. Cho SH, Hall IP, Wheatley A, Dewar J, Abraha D, Del Mundo J, Lee H, Oh CK: **Possible role of the 4G/5G polymorphism of the plasminogen activator inhibitor 1 gene in the development of asthma**. *J Allergy Clin Immunol* 2001, **108**(2):212-214.

46. Suttner K, Rosenstiel P, Depner M, Schedel M, Pinto LA, Ruether A, Adamski J, Klopp N, Illig T, Vogelberg C, Schreiber S, von Mutius E, Kabesch M: **TBX21 gene variants increase childhood asthma risk in combination with HLX1 variants**. *J Allergy Clin Immunol* 2009, **123**(5):1062-1068, 1068 e1061-1068.

47. Munthe-Kaas MC, Carlsen KH, Haland G, Devulapalli CS, Gervin K, Egeland T, Carlsen KL, Undlien D: **T cell-specific T-box transcription factor haplotype is associated with allergic asthma in children**. *J Allergy Clin Immunol* 2008, **121**(1):51-56.

48. Raby BA, Hwang ES, Van Steen K, Tantisira K, Peng S, Litonjua A, Lazarus R, Giallourakis C, Rioux JD, Sparrow D, Silverman EK, Glimcher LH, Weiss ST: **T-bet polymorphisms are associated with asthma and airway hyperresponsiveness**. *Am J Respir Crit Care Med* 2006, **173**(1):64-70.

49. Moller-Larsen S, Nyegaard M, Haagerup A, Vestbo J, Kruse TA, Borglum AD: **Association analysis identifies TLR7 and TLR8 as novel risk genes in asthma and related disorders**. *Thorax* 2008, **63**(12):1064-1069.

50. Saadi A, Gao G, Li H, Wei C, Gong Y, Liu Q: **Association study between vitamin D receptor gene polymorphisms and asthma in the Chinese Han population: a case-control study**. *BMC Med Genet* 2009, **10**:71.

51. Daley D, Lemire M, Akhabir L, Chan-Yeung M, He JQ, McDonald T, Sandford A, Stefanowicz D, Tripp B, Zamar D, Bosse Y, Ferretti V, Montpetit A, Tessier MC, Becker A, Kozyrskyj AL, Beilby J, McCaskie PA, Musk B, Warrington N, James A, Laprise C, Palmer LJ, Pare PD, Hudson TJ: **Analyses of associations with asthma in four asthma population samples from Canada and Australia**. *Hum Genet* 2009, **125**(4):445-459.

52. Minelli C, Granell R, Newson R, Rose-Zerilli MJ, Torrent M, Ring SM, Holloway JW, Shaheen SO, Henderson JA: **Glutathione-S-transferase genes and asthma phenotypes: a Human Genome Epidemiology (HuGE) systematic review and meta-analysis including unpublished data**. *Int J Epidemiol* 2010, **39**(2):539-562.

53. Movahedi M, Moin M, Gharagozlou M, Aghamohammadi A, Dianat S, Moradi B, Nicknam MH, Nikbin B, Amirzargar A: **Association of HLA class II alleles with childhood asthma and Total IgE levels**. *Iran J Allergy Asthma Immunol* 2008, **7**(4):215-220.

54. Munthe-Kaas MC, Carlsen KL, Carlsen KH, Egeland T, Haland G, Devulapalli CS, Akselsen H, Undlien D: **HLA Dr-Dq haplotypes and the TNFA-308 polymorphism: associations with asthma and allergy**. *Allergy* 2007, **62**(9):991-998.

55. Lee CC, Lin WY, Wan L, Tsai Y, Tsai CH, Huang CM, Chen CP, Tsai FJ: **Association of interleukin-18 gene polymorphism with asthma in Chinese patients**. *J Clin Lab Anal* 2008, **22**(1):39-44.

56. Imboden M, Nicod L, Nieters A, Glaus E, Matyas G, Bircher AJ, Ackermann-Liebrich U, Berger W, Probst-Hensch NM: **The common G-allele of interleukin-18 single-nucleotide polymorphism is a genetic risk factor for atopic asthma. The SAPALDIA Cohort Study**. *Clin Exp Allergy* 2006, **36**(2):211-218.

57. Higa S, Hirano T, Mayumi M, Hiraoka M, Ohshima Y, Nambu M, Yamaguchi E, Hizawa N, Kondo N, Matsui E, Katada Y, Miyatake A, Kawase I, Tanaka T: **Association between interleukin-18 gene polymorphism 105A/C and asthma**. *Clin Exp Allergy* 2003, **33**(8):1097-1102.

58. Martinez B, Barrios K, Vergara C, Mercado D, Jimenez S, Gusmao L, Caraballo L: **A NOS1 gene polymorphism associated with asthma and specific immunoglobulin E response to mite allergens in a Colombian population**. *Int Arch Allergy Immunol* 2007, **144**(2):105-113.

59. Hossny EM, Amr NH, Elsayed SB, Nasr RA, Ibraheim EM: **Association of polymorphisms in the mast cell chymase gene promoter region (-1903 g/A) and (TG)n(GA)m repeat downstream of the gene with bronchial asthma in children**. *J Investig Allergol Clin Immunol* 2008, **18**(5):376-381.

60. Masumoto J, Yang K, Varambally S, Hasegawa M, Tomlins SA, Qiu S, Fujimoto Y, Kawasaki A, Foster SJ, Horie Y, Mak TW, Nunez G, Chinnaiyan AM, Fukase K, Inohara N: **Nod1 acts as an intracellular receptor to stimulate chemokine production and neutrophil recruitment in vivo**. *J Exp Med* 2006, **203**(1):203-213.

61. Pegorier S, Arouche N, Dombret MC, Aubier M, Pretolani M: **Augmented epithelial endothelin-1 expression in refractory asthma**. *J Allergy Clin Immunol* 2007, **120**(6):1301-1307.

62. Nagase T, Kurihara H, Kurihara Y, Aoki-Nagase T, Nagai R, Ouchi Y: **Disruption of ET-1 gene enhances pulmonary responses to methacholine via functional mechanism in knockout mice**. *J Appl Physiol* 1999, **87**(6):2020-2024.

63. Chatila TA: **Interleukin-4 receptor signaling pathways in asthma pathogenesis**. *Trends Mol Med* 2004, **10**(10):493-499.

64. Jetten AM: **Retinoid-related orphan receptors (RORs): critical roles in development, immunity, circadian rhythm, and cellular metabolism**. *Nucl Recept Signal* 2009, **7**:e003.

65. Jaradat M, Stapleton C, Tilley SL, Dixon D, Erikson CJ, McCaskill JG, Kang HS, Angers M, Liao G, Collins J, Grissom S, Jetten AM: **Modulatory role for retinoid-related orphan receptor alpha in allergen-induced lung inflammation**. *Am J Respir Crit Care Med* 2006, **174**(12):1299-1309.

66. Chu EK, Cheng J, Foley JS, Mecham BH, Owen CA, Haley KJ, Mariani TJ, Kohane IS, Tschumperlin DJ, Drazen JM: **Induction of the plasminogen activator system by mechanical stimulation of human bronchial epithelial cells**. *Am J Respir Cell Mol Biol* 2006, **35**(6):628-638.

67. Matsuzaki S, Ishizuka T, Hisada T, Aoki H, Komachi M, Ichimonji I, Utsugi M, Ono A, Koga Y, Dobashi K, Kurose H, Tomura H, Mori M, Okajima F: **Lysophosphatidic acid inhibits CC chemokine ligand 5/RANTES production by blocking IRF-1-mediated gene transcription in human bronchial epithelial cells**. *J Immunol* 2010, **185**(8):4863-4872.

68. Berlin AA, Lincoln P, Tomkinson A, Lukacs NW: **Inhibition of stem cell factor reduces pulmonary cytokine levels during allergic airway responses**. *Clin Exp Immunol* 2004, **136**(1):15-20.

69. McKay A, Komai-Koma M, MacLeod KJ, Campbell CC, Kitson SM, Chaudhuri R, Thomson L, McSharry C, Liew FY, Thomson NC: **Interleukin-18 levels in induced sputum are reduced in asthmatic and normal smokers**. *Clin Exp Allergy* 2004, **34**(6):904-910.

70. Yamagata S, Tomita K, Sato R, Niwa A, Higashino H, Tohda Y: **Interleukin-18-deficient mice exhibit diminished chronic inflammation and airway remodelling in ovalbumin-induced asthma model**. *Clin Exp Immunol* 2008, **154**(3):295-304.

71. Goenka S, Kaplan MH: **Transcriptional regulation by STAT6**. *Immunol Res* 2011, **50**(1):87-96.

72. Yang CJ, Liu YK, Liu CL, Shen CN, Kuo ML, Su CC, Tseng CP, Yen TC, Shen CR: **Inhibition of acidic mammalian chitinase by RNA interference suppresses ovalbumin-sensitized allergic asthma**. *Hum Gene Ther* 2009, **20**(12):1597-1606.

73. Zheng XQ, Li CC, Xu DP, Lin A, Bao WG, Yang GS, Yan WH: **Analysis of the plasma soluble human leukocyte antigen-G and interleukin-10 levels in childhood atopic asthma**. *Hum Immunol* 2010, **71**(10):982-987.

74. Chen YQ, Shi HZ: **CD28/CTLA-4--CD80/CD86 and ICOS--B7RP-1 costimulatory pathway in bronchial asthma**. *Allergy* 2006, **61**(1):15-26.

75. Crosby JR, Guha M, Tung D, Miller DA, Bender B, Condon TP, York-DeFalco C, Geary RS, Monia BP, Karras JG, Gregory SA: **Inhaled CD86 antisense oligonucleotide suppresses pulmonary inflammation and airway hyper-responsiveness in allergic mice**. *J Pharmacol Exp Ther* 2007, **321**(3):938-946.

76. Madore AM, Perron S, Turmel V, Laviolette M, Bissonnette EY, Laprise C: **Alveolar macrophages in allergic asthma: an expression signature characterized by heat shock protein pathways**. *Hum Immunol* 2010, **71**(2):144-150.

77. Kowal K, Moniuszko M, Zukowski S, Bodzenta-Lukaszyk A: **Concentrations of plasminogen activator inhibitor-1 (PAI-1) and urokinase plasminogen activator (uPA) in induced sputum of asthma patients after allergen challenge**. *Folia Histochem Cytobiol* 2010, **48**(4):518-523.

78. Kucharewicz I, Mogielnicki A, Kasacka I, Buczko W, Bodzenta-Lukaszyk A: **Plasmin system regulation in an ovalbumin-induced rat model of asthma**. *Int Arch Allergy Immunol* 2008, **147**(3):190-196.

79. Berry MA, Hargadon B, Shelley M, Parker D, Shaw DE, Green RH, Bradding P, Brightling CE, Wardlaw AJ, Pavord ID: **Evidence of a role of tumor necrosis factor alpha in refractory asthma**. *N Engl J Med* 2006, **354**(7):697-708.

80. Bailey MT, Kierstein S, Sharma S, Spaits M, Kinsey SG, Tliba O, Amrani Y, Sheridan JF, Panettieri RA, Haczku A: **Social stress enhances allergen-induced airway inflammation in mice and inhibits corticosteroid responsiveness of cytokine production**. *J Immunol* 2009, **182**(12):7888-7896.

81. Ye Q, Fujita M, Ouchi H, Inoshima I, Maeyama T, Kuwano K, Horiuchi Y, Hara N, Nakanishi Y: **Serum CC-10 in inflammatory lung diseases**. *Respiration* 2004, **71**(5):505-510.

82. Ray R, Choi M, Zhang Z, Silverman GA, Askew D, Mukherjee AB: **Uteroglobin suppresses SCCA gene expression associated with allergic asthma**. *J Biol Chem* 2005, **280**(11):9761-9764.
